# Supplementary material for: Enhancing georeferenced biodiversity inventories: automated information extraction from literature records reveal the gaps
Source: PeerJ. 2022 Aug 18;10:e13921. doi: 10.7717/peerj.13921 (PMC9393005; doi:10.7717/peerj.13921)
Supplement: Supplemental Information 1 [file peerj-10-13921-s001.pdf]

Appendix S1: Extended Methods

Supporting information to: Enhancing georeferenced biodiversity inventories: automated information extraction from literature records reveal the gaps

Bjørn Tore Kopperud[1], Scott Lidgard[2], Lee Hsiang Liow[1,3]

[1]*Natural History Museum, University of Oslo, PO Box 1172 Blindern, 0318 Oslo, Norway*
[2]*Negaunee Integrative Research Center, Field Museum, 1400 South Lake Shore Drive, Chicago IL, 60605, U.S.A*
[3]*Centre for Ecological and Evolutionary Synthesis, Department of Biosciences, University of Oslo, PO Box 1066 Blindern, 0316 Oslo, Norway*

OBIS and GBIF data

We used the R-package robis (Provoost and Bosch, 2021) to download OBIS data for cheilostome bryozoans with the function call ‘robis::occurrence(scientificname = ”Cheilostomatida”)’. We used the web interface to access GBIF, by querying for occurrence records under the order ”Cheilostomatida” (dataset doi: 10.15468/dl.58pd9h). We accessed both databases on 21.02.2022. We considered only records with Linnéan binomial names, i.e., containing a genus name and a species name. See the main text for how we treated taxonomic uncertainties. We did not vet the OBIS or GBIF data (nor the TMO data described in the next section) for errors using manual case-by-case inspection.

Automated retrieval of location-species data

Our text-mined occurrences (TMO) are retrieved in a series of steps as already summarized in the main text. The steps, detailed below, are as follows. (i) We acquire the relevant literature. (ii) We annotate the text with linguistic features, such as recognizing genus or species names (henceforth taxon or taxa) and toponyms (i.e. place names) as entities. (iii) We extract all candidates: pairs of taxon names and toponyms that co-occur in a sentence. (iv) We build a machine-learning classifier that verifies that the candidates have correct toponyms. (v) We develop a machine-learning model that classifies each candidate according to whether or not the sentence implies that the taxon is found in the location described by the toponym. (vi) We use a geocoding service to pin the toponyms to a location.

(i) Acquisition of literature

For the TMO, we used two different corpora (i.e., collections) of published works. In an earlier publication (Kopperud et al., 2019), we used a corpus that was primarily focused on paleontological literature. We supplemented that corpus with publications that were likely to contain descriptions of extant cheilostomes, such as ecological surveys or taxonomic descriptions of new or revised taxa. This first corpus (our own collection as indicated in the main text) consists of 3223 published documents with associated pdfs. Of these, we filtered those that were not in English using a language detection tool (Nakatani, 2010), retaining about 80% of the extracted publications. The second corpus we used was extracted from the GeoDeepDive or GDD archive (www.geodeepdive.org). GDD is a (U.S.) National Science Foundation funded project that gathers full-text contents of journal articles to facilitate data-mining. We searched all documents available on GDD, in Mar 2019 (when more than 10 million documents were available), and selected publications containing any cheilostome genus name found in www.bryozoa.net (Bock, 2020) and/or World Register of Marine Species (Worms Editorial Board, 2022). From these selected documents, we extract sentences that contain potential candidate pairs (see later section),

that we then filter to remove non-English language ones. Intellectual property rights restrict GDD from redistributing documents in their entirety. Thus, while we could run our text-mining procedure on GDD and retrieve a summary of candidates (explained later), we were not able to 'read' the main text. For this reason, we filtered non-English literature using the same language detection tool (Nakatani, 2010) but on the candidate level, instead of the document level.

Since our corpora consist of both paleontological and recent ecological or taxonomic literature, we sought to eliminate documents of the former. We used a machine-learning classifier as, first, there are too many articles to label, and second, since we did not have access to 'read' the documents in GDD as explained above. We manually annotated 839 publications being primarily about fossils, and 825 primarily about recent faunas. Using these annotations, we trained a supervised text classifier to predict if the document in question is either more about fossil or recent faunas. Specifically, we trained a support vector machine (SVM), implemented in scikit-learn (Pedregosa et al., 2011). We used a radial-basis kernel function (allowing for a non-linear decision boundary) for the SVM, but otherwise used default settings. To enable this classifier, we used the number of mentions of geologic time intervals (e.g. "Upper Eocene") and stratigraphic names (e.g. "Needmore Shale", or "Lincolnshire Limestone") per document as features (see the next section for how these are recognized). We evaluated the classifier using 5-fold cross validation and achieved an accuracy of 83.4 $\pm$ 1.8% (standard deviation). To counteract possible effects of class imbalance during cross validation, we weighted the inputs such that they are inversely proportional to their class frequency during training. We then applied the classifier to any documents that were not yet labeled, and after filtering we retained 58% of publications pertaining mainly to recent faunas.

(ii) Linguistic annotation

We used CoreNLP (Manning et al., 2014) for an initial natural language analysis, including tokenization, named-entity recognition, and dependency grammar annotation. Tokenization splits text into tokens, which are words and punctuation, after which sentences can be demarcated. In order to recognize bryozoan species names, we first assembled a list of known species names, based on the online compendia www.bryozoa.net and WoRMS. In order to recognize and count mentions of geologic time intervals and stratigraphic names, we used known names from GeoWhen (Rohde, 2022) and Macrostrat (Peters et al., 2018b). We used these names to assemble a set of rules (TokensRegex, Chang and Manning, 2014) that recognize relevant names of species, stratigraphic units and geological time intervals. Finally, we applied CoreNLP to assign dependency grammar (i.e. relations among words in a sentence, De Marneffe et al. 2014), which we used as a feature for the relation classifier explained later. We used a generic, pre-trained machine-learning model (Finkel et al., 2005) to recognize toponyms (i.e. place names or locations) in text. We found that this generic named-entity recognition was prone to detect false positives such as author names (e.g. "Hincks" or "Darwin"). In order to remedy this, we trained a machine-learning

classifier to verify whether the tagged entities were indeed proper toponyms (see section "Toponym verification").

(iii) Candidate extraction

This was largely detailed in the main text, with the sentence below as an illustration (Tilbrook et al., 2001, page 50):

> "The avicularia resemble those seen in *B. intermedia* (Hincks, 1881b), from Tasmania and New Zealand, but this species is only just over half the size of *B. cookae*."

Whenever we found an abbreviated genus name, such as "*B.*", we searched for genus names in the current and 14 previous sentences. In reverse chronological order, we looked for any span (i.e. one or more consecutive tokens) tagged as being a taxon that starts with the same capital letter, and chose the first genus name for the de-abbreviation (here "*Beania*").

(iv) Toponym verification

As mentioned in section (ii) the generic named-entity recognition was prone to detect false positives. To reduce false positives here, we evaluated 6000 candidates and annotated whether the assigned toponym was indeed a toponym or a false positive. We used these annotations to train a machine-learning classifier; a neural network implemented in Keras (Chollet et al., 2015). For the previously mentioned classifiers (see sections i,ii), we used reference implementations and did not change the default settings considerably. For the toponym classifier, however, we made several decisions when engineering the classifier. For this reason, we detail the toponym classifier more closely than the other machine-learning models we have used.

A neural network can be thought of as a computational graph, with inputs, outputs, and several operations in the middle. For organizational purposes, we refer to sections of these operations as "layers". Each layer has a set of associated coefficients or weights that are either set a priori or initialized randomly. For a set of inputs and labels, it is possible to evaluate a loss function that estimates how much the output diverges from the assigned label. Next, one can employ a back-propagation technique (Rumelhart et al., 1986) to compute partial derivatives or gradients for the loss value with respect to each coefficient in the network. These gradients, coupled with an algorithm for gradient descent, can be used to learn the coefficients, and consequently minimize the loss of the network during training. We split the annotation data in three parts: training-, validation- and test sets (roughly 80, 10, 10% candidates per set). We used the training data to learn the coefficients of the network. We used the validation data to fit the hyperparameters (e.g. learning rate, layer dimensions) and to decide when to stop training. By evaluating the trained network on the test data we get an estimate of how well the classifier performs on out-of-sample

sentences. In order to remove possible confounding effects of similar sentences in a publication, we made sure that all candidates from a single publication were contained within only one of the training, validation or test datasets.

Our toponym classifier received two inputs: the sequence of tokens in the candidate's sentence, and a sequence of indicator variables denoting where the toponym is in the sentence. These were fed to embedding layers: the tokens were embedded in a 300-dimensional vector space using a pre-trained fastText model (Bojanowski et al., 2017), and the indicator variables were embedded as two orthogonal unit-vectors. The coefficients for the embedding layers were constrained to be static during training. After concatenation, we fed the embedded values to a Long Short-Term Memory (LSTM, Hochreiter and Schmidhuber 1997) recurrent layer, with dropout values of 0.2. Since the sentences were of variable length, we padded the shorter sentences and used masking to avoid any processing of the padded features during training. We used the final time step of the second recurrent layer as input to a final two-dimensional hidden layer, with a softmax activation function. Since the softmax function maps all real inputs to outputs of [0,1], while ensuring that they sum to one, we interpret the output as a probability mass. We used cross entropy as the loss function, and the ADAM algorithm (Kingma and Ba, 2014) for gradient descent. Unless otherwise stated, we used default settings in Keras for other parameters and options.

For each epoch (an iteration in which the classifier sees the entire training set), we computed the F1 metric (the harmonic mean of precision and recall). We trained the classifier for 50 epochs, and saved the coefficients whenever the validation F1 was better than the previously best validation F1, effectively conditioning the learning on maximizing the F1 metric. The toponym classifier achieved an F1 of 94.7%, accuracy of 93.2%, recall of 97.4%, precision of 92.2%, and a false positive rate of 14.0% (see Fig. S6a) when evaluated on the test set.

(v) Relation classifier

We manually annotated 4938 unique candidates (species-toponym pairs) to form our training dataset. If the sentence explicitly stated or strongly implied that the taxon was found in the mentioned location, we labelled the candidate as 'positive'. If not, we labelled the candidate as 'negative'. These annotations were made by two persons, with intra- and inter-annotator accuracies of 91% (n = 200) and 85.8% (n = 211), respectively. This classifier is analogous to the toponym classifier (see previous section), except we use neither the indicator variables, nor the entire sentence. Instead, we use the sequence of tokens along shortest path in dependency grammar between the two spans in the sentence (see Xu et al., 2015; Kopperud et al., 2019). We trained our machine-classifier using the training set of these to evaluate the classifier. This relation classifier achieved an F1 of 76.8%, accuracy of 73.1%, precision of 74.8%, recall of 78.9% and false positive rate of 34.3% (see Fig. S6b).

We discussed in the main text how our estimated false positive rate of 34.3% is better than a random classifier baseline, but not as good as the false positive

rates between human annotators at 14% and 16% (n = 200, assuming annotator A is correct, then evaluating annotator B, and vice versa). Similarly, the classifier accuracy of 73.1% is better than a weighted coin random classifier. If we assume this random classifier is as unbalanced as the labelled candidates (60% positives), this classifier gives an accuracy baseline of 52% ($0.6^2 + 0.4^2 = 0.52$). Yet, this is not as good as the intra- and inter-annotator accuracies at 91% (n = 200) and 85.6% (n = 211), respectively. Incidentally, applying the relation classifier from Kopperud et al. (2019) trained on relating taxon spans and geologic time interval spans, yielded virtually the same performance (evaluated on the same test data), despite not being trained on the specific task or having 'seen' any toponym spans.

The relation classifier could potentially be improved by increasing the amount of training data, and/or using a more complex classifier, such as taking into account context-dependent word embeddings (Peters et al., 2018a; Devlin et al., 2019). However, we believe that the major bottleneck is not lack of natural language understanding. Rather, the candidates themselves are not always linguistically sound, coherent and self-contained sentences. Specifically, much of the information that relays the relation between the two spans in the taxonomic literature of interest is coded in titles, sub-titles, variation in font type, font size, and spatial layout of the paragraphs. This introduces errors for the sentence splitting procedure in CoreNLP. The features we used to capture the relation (Part-of-Speech and Dependency grammar) are inherently limited since they are designed to work on relatively coherent, self-contained and complete sentences. Standard Natural Language Processing tools are flexible and relatively easy to adopt, but for this particular problem it could be advantageous to use other, non-linguistic features to facilitate the information extraction, as has been suggested in the knowledge base creation literature (e.g. Schlichtkrull et al., 2018) but is outside the scope of our study.

(vi) Geocoding: converting toponyms to coordinates

As stated in the main text, we used the Google geocoding service (`https://developers.google.com/maps/documentation/geocoding/`) to acquire a bounding box with four latitude-longitude coordinates and a centroid (Fig. S4). For any query, the geocoding service returns one record that best matches the toponym. Some toponyms are inherently more precise than others (e.g., "Suez Canal" vs "Red Sea"), and the service also provides a description of how precise it infers the record to be. These description codes are "ROOFTOP", "RANGE_INTERPOLATED", "GEOMETRIC_CENTER", and "APPROXIMATE", ranging from very fine to coarse spatial resolution. Our toponyms are in general not as precise as the most precise locations returned by the geocoding service. For our data, such precise matches are usually incorrect mappings of a toponym to a location (e.g. "Norfolk Ridge", a marine ridge between New Zealand and New Caledonia is mapped to a street address in Norfolk, USA). To avoid such problems, we only used the categories "GEOMETRIC_CENTER" and "APPROXIMATE" for the geocoder results, retaining 88.3% of the unique

locations. Despite considering only these coarse categories, the spatial resolutions of the toponyms are highly variable. To get an estimate of whether the resulting records were precise or imprecise, we calculated and used the area of the bounding box as a proxy for spatial precision. We removed mappings that did not have an associated bounding box (24.3%). We assumed that the shape of the Earth can reasonably be approximated by that of a sphere, and calculated the area of the bounding box as follows:

$$A = 2\pi r^2(\sin(\alpha_{\text{north}}) - \sin(\alpha_{\text{south}}))(\beta_{\text{east}} - \beta_{\text{west}})/360°, \tag{S1}$$

where r is the radius of the earth, α are the latitudes and β the longitudes of the bounding edges. The area calculation assumes that the planes demarcating the northern and southern edges are perpendicular to the axis of rotation, and that the planes demarcating the western and eastern edges are coincident with the axis of rotation. The bounding box area is always larger than the actual area of the location described by the toponym. For example, the "Mediterranean" or "Mediterranean Sea" is in reality about 2 500 000 km^2 (Salah and Boxer, 2022) but our bounding box estimate is about 6 350 000 km^2. We used these area estimates to remove species occurrence records in locations represented by bounding boxes that were larger than a certain threshold. For the analyses presented in the main text, the threshold was 1.86% of the Earth's surface (see Fig. S5).

Regional maps

In order to separate richness estimations for the different ocean basins (Fig. 3), we drew polygons (using `www.geojson.io`) to delimit ocean basins and partitions thereof. The coordinates and plots for the ocean polygons can be found in the code supplement under /data/regions.

Figures

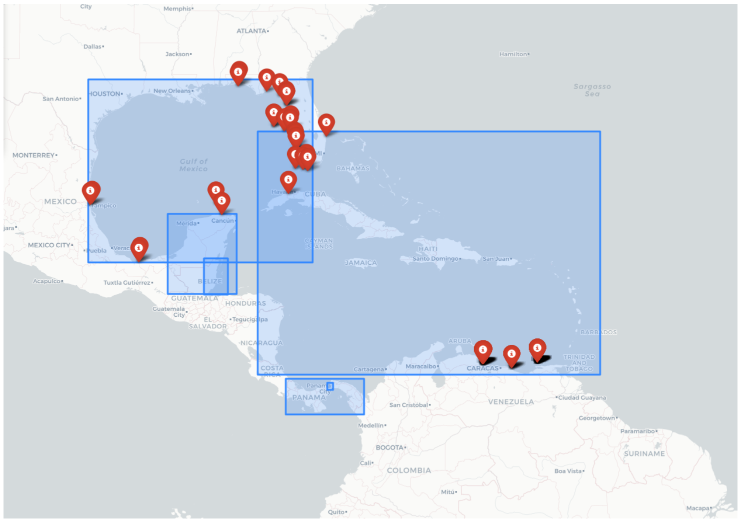

Figure S1: An example of bounding boxes and OBIS and GBIF records. The red balloons indicate database coordinates for *Schizoporella pungens* and blue rectangles the TMO bounding boxes for toponyms associated with *S. pungens*. The toponyms are "Pacific Panama", "Belize", "Panama Canal", "Yucatan Peninsula", "Gulf of Mexico" and "Caribbean" (the largest blue box on this figure). Another toponym included in the TMO data for *S. pungens* is "Fort Pierce Inlet" but it is too small to be visible here. Not shown is the location "Rio de Janeiro", a TMO false positive, but there are two OBIS records from Okinawa, Japan, corroborating evidence that *S. pungens* is a ship-borne invading species (McCann et al., 2019).

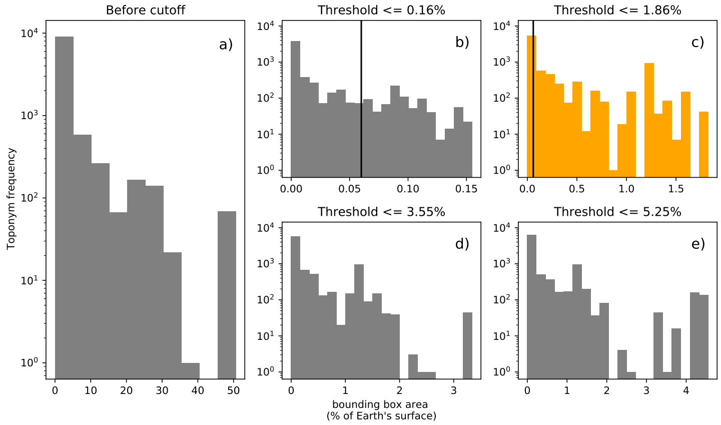

Figure S2: Toponym bounding box size distributions. a) shows the distribution prior to filtering, and b)-e) show the distribution after filtering records with bounding boxes larger than areas 0.16, 1.86, 3.55 and 5.25, in units of percentage of the Earth's surface, respectively. 81.9% of all TMO records are included in c), where 1.86% is the threshold that is used for the figures in the main text. Of all TMO records in c), 54% are smaller than a 5° latitude by 5° longitude bin at the equator (area 0.06% of the Earth's surface), indicated by the black vertical line. The frequencies are not unique toponyms, e.g. two mentions of "Adriatic Sea" results in two counts in these figures.

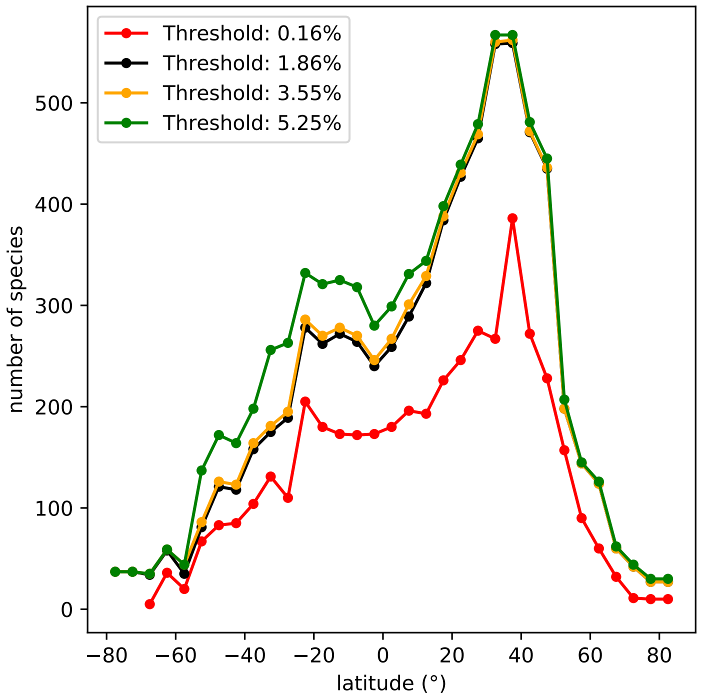

Figure S3: TMO range-through species richness with varying thresholds for removing toponym locations. The thresholds are in units of percentage of the Earth's surface. The latitudinal bands are 5° and equiangular. The analyses represented in the main text are based on the 1.86% threshold (black line). See also Fig. S2.

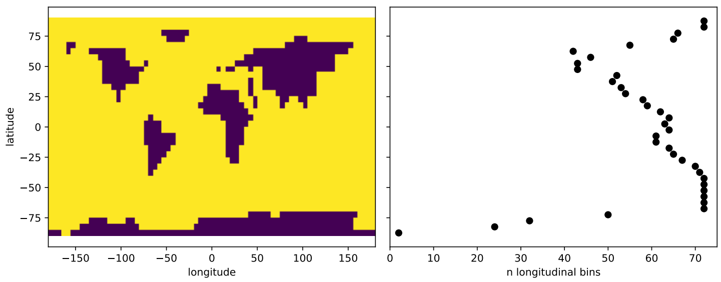

Figure S4: Landlocked areas. a) shows 5° by 5° land-locked bins as derived from a 1:10m coastlines map (map data: Patterson, 2019), and b) the distribution of the resulting valid longitudinal sampling bins across latitude.

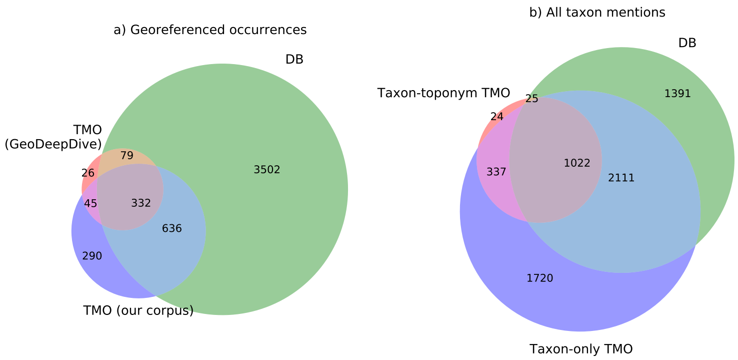

Figure S5: Species overlap between TMO and OBIS/GBIF records. The Venn diagrams show species counts for a) the occurrences we were able to geo-reference, and b) all taxon mentions, regardless of geo-referencing. Taxon-toponym TMO refers to taxon mentions from the candidates discussed in the main text, while Taxon-only refers to all text-mined mentions of any bryozoan species with accepted genus names in the published literature. We were not able to retrieve taxon-only mentions from GeoDeepDive. Note that for b), the taxon-only TMO species counts sum to 5190, and the total is 6630. These numbers are higher than the figures described by (Bock and Gordon, 2013), and (Gordon et al., 2019), however all of our species names are considered accepted according to (WoRMS Editorial Board, 2022). The counts are slightly inflated according to the most recent bryozoan species tally, however the figure still gives an impression of how well the cheilostome taxonomic inventory is represented in TMO and OBIS/GBIF records.

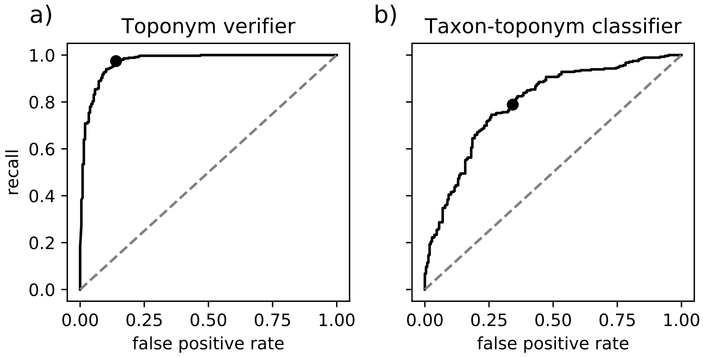

Figure S6: Classifier receiver operating characteristic (ROC) curves. a) shows the ROC for the toponym verification classifier. The black line is the performance of the toponym verifier evaluated on the test set (n = 557), and the dashed line is the expected performance for a random classifier. The standard decision boundary of 0.5 (black point) yields a false positive rate of 14% and recall of 97%. b) shows the equivalent for the taxon-toponym relation classifier (false positive rate of 34%, recall of 79%, n = 495).

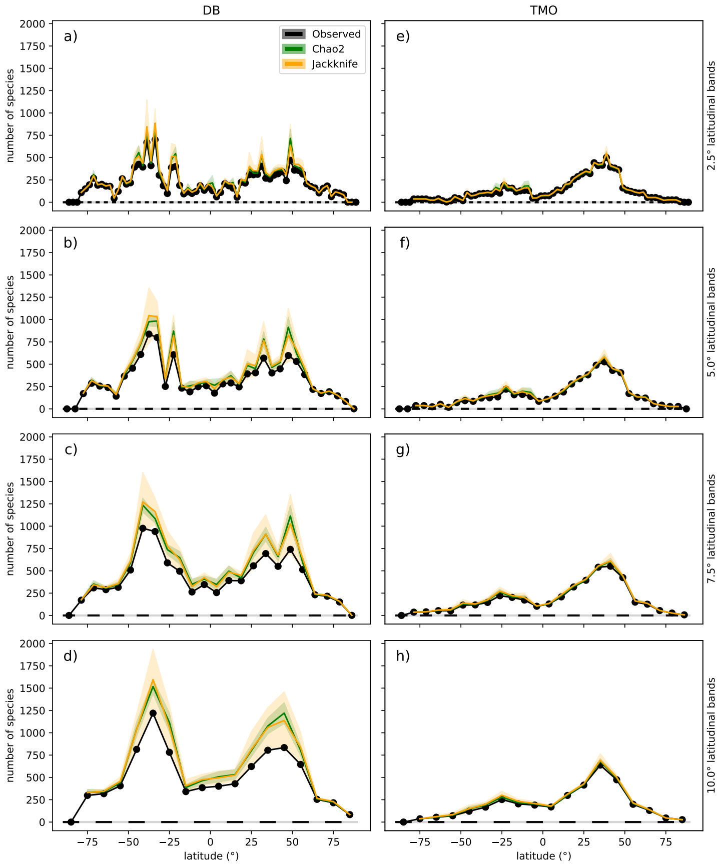

Figure S7: Species richness estimates with varying latitudinal bands. Estimates of Chao2 and Jackknife species richness with equiangular latitudinal bands that are 2.5°, 5° (identical to main text Fig. 2a,b), 7.5° and 10.0°. Panels a)-d) use OBIS/GBIF data, while e)-f) use TMO data. All panels are conditional on 5.0° longitudinal sampling bins.

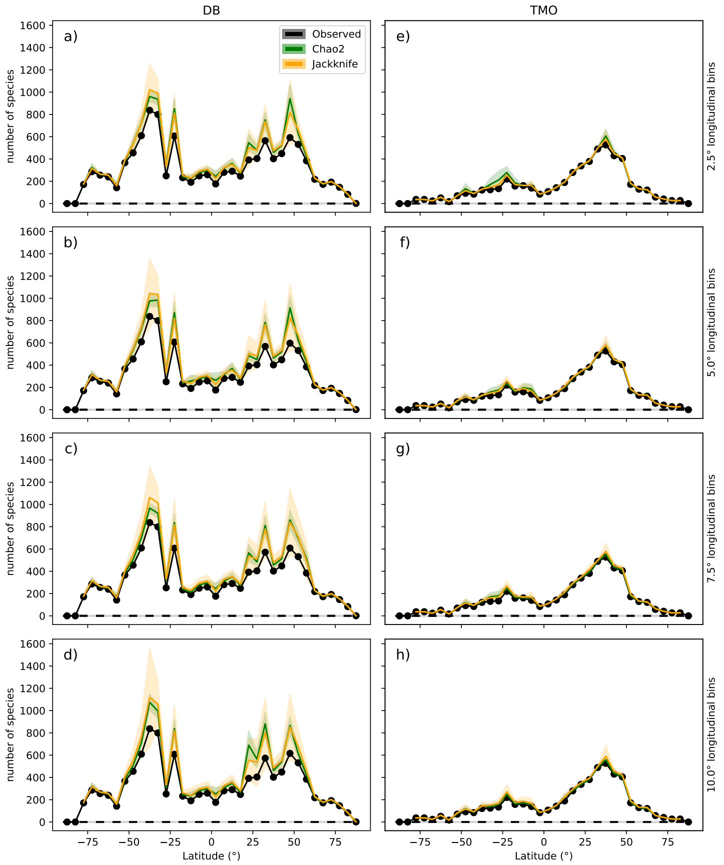

Figure S8: Species richness estimates with varying longitudinal sampling bins. Estimates of Chao2 and Jackknife species richness with longitudinal sampling bins that are 2.5°, 5° (identical to main text Fig. 2a,b), 7.5° and 10°. Panels a)-d) use OBIS/GBIF data, while e)-h) use TMO data. All panels are conditional on 5° equiangular latitudinal bands.

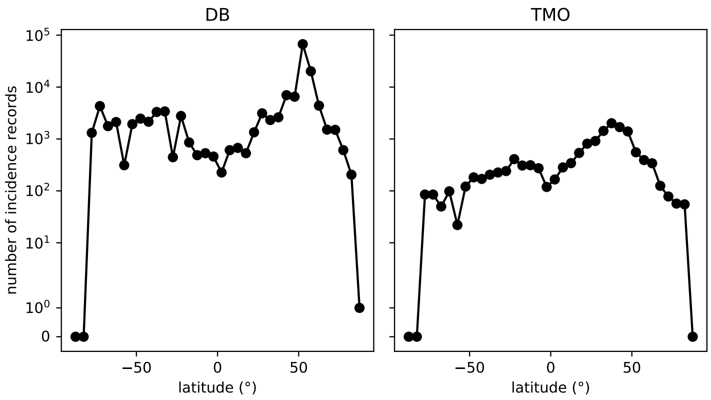

Figure S9: Number of incidence records across latitude. Each point represents data from a 5° latitudinal band. The y-axis is linear between 0 and 1, and log-transformed for $y > 1$.

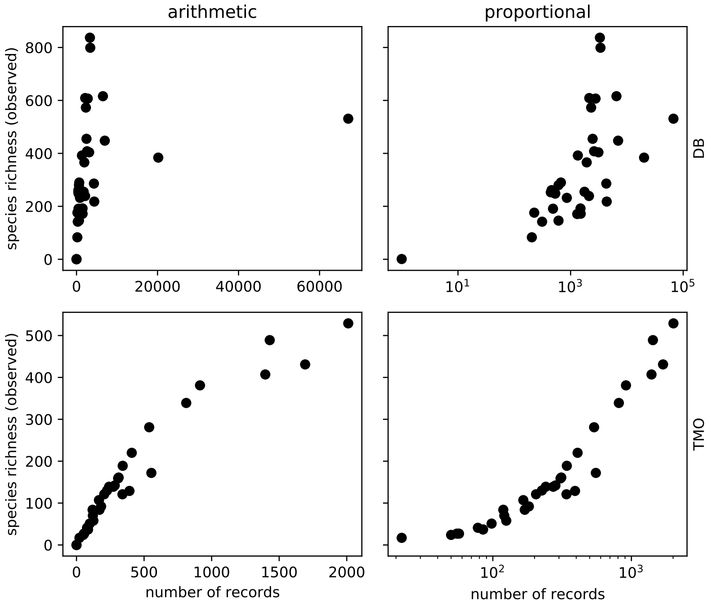

Figure S10: Species richness versus the number of records. Each point represents data from a 5° latitudinal band. The species richness estimates are the observed species counts, corresponding with the black lines in Fig. 2a,b. Bands with zero entries are omitted. Both rows represent the same data, however the x-axis is log-transformed in the right column.

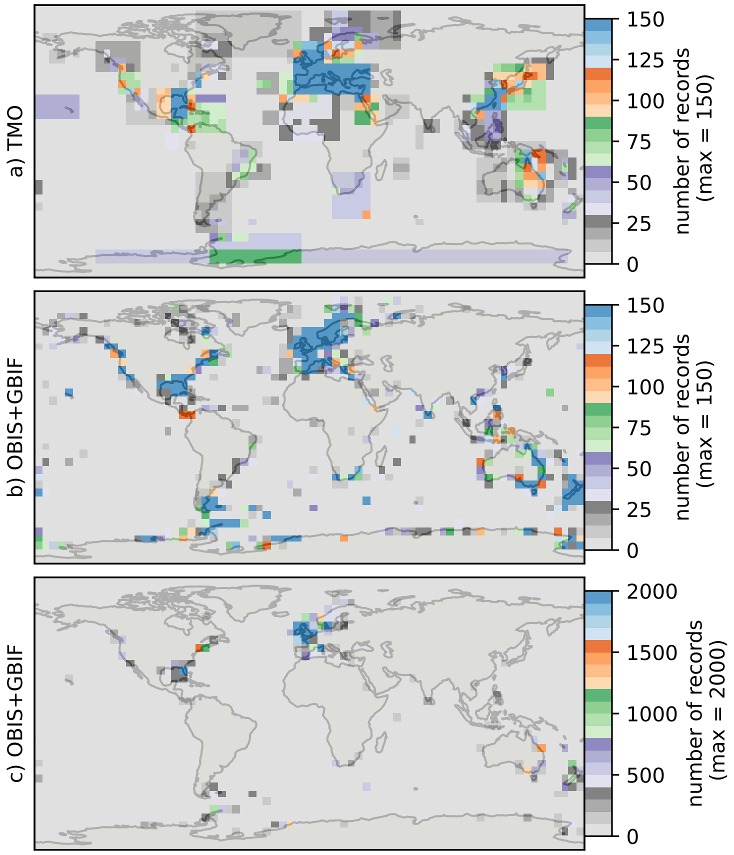

Figure S11: Heatmaps for cheilostome bryozoan occurrence records per 5° latitude by 5° longitude bins. The color axes are truncated for visualization purposes, to a maximum of 200, 200 and 2000 in a), b), c), respectively. There are about 750 maximum records per bin in the Mediterranean for the text-mined occurrences (TMO), about 79000 maximum records in the British Isles for the two combined databases (DB = OBIS and GBIF). The globe is plotted using the plate carrée projection, but otherwise equal to Fig. 4.

References

Bock, P. E. and Gordon, D. P. (2013). Phylum Bryozoa Ehrenberg, 1831. *Zootaxa*, 3703(1):67–74.

Bojanowski, P., Grave, E., Joulin, A., and Mikolov, T. (2017). Enriching word vectors with subword information. *Transactions of the Association for Computational Linguistics*, 5:135–146.

Chang, A. X. and Manning, C. D. (2014). Tokensregex: Defining cascaded regular expressions over tokens. *Stanford University Computer Science Technical Reports. CSTR*, 2:2014.

Chollet, F. et al. (2015). Keras: The python deep learning library. `https://keras.io`.

De Marneffe, M.-C., Dozat, T., Silveira, N., Haverinen, K., Ginter, F., Nivre, J., and Manning, C. D. (2014). Universal Stanford dependencies: A cross-linguistic typology. In *LREC*, volume 14, pages 4585–4592.

Devlin, J., Chang, M.-W., Lee, K., and Toutanova, K. (2019). BERT: Pre-training of deep bidirectional transformers for language understanding. In *Proceedings of the 2019 Conference of the North American Chapter of the Association for Computational Linguistics: Human Language Technologies, Volume 1 (Long and Short Papers)*, pages 4171–4186, Minneapolis, Minnesota. Association for Computational Linguistics.

Finkel, J. R., Grenager, T., and Manning, C. D. (2005). Incorporating non-local information into information extraction systems by gibbs sampling. In *Proceedings of the 43rd Annual Meeting of the Association for Computational Linguistics (ACL'05)*, pages 363–370.

Gordon, D. P., Bock, P. E., Souto-Derungs, J., and Reverter-Gil, O. (2019). A bryozoan tale of two continents: faunistic data for the Recent Bryozoa of Greater Australia (Sahul) and Zealandia, with European comparisons. *Australasian Palaeontological Memoirs*, pages 13–22.

Hochreiter, S. and Schmidhuber, J. (1997). Long short-term memory. *Neural computation*, 9(8):1735–1780.

Kingma, D. P. and Ba, J. (2014). Adam: A method for stochastic optimization. *arXiv preprint arXiv:1412.6980*.

Kopperud, B. T., Lidgard, S., and Liow, L. H. (2019). Text-mined fossil biodiversity dynamics using machine learning. *Proceedings of the Royal Society B*, 286(1901):20190022.

Manning, C. D., Surdeanu, M., Bauer, J., Finkel, J. R., Bethard, S., and McClosky, D. (2014). The stanford corenlp natural language processing toolkit. In *Proceedings of 52nd annual meeting of the association for computational linguistics: system demonstrations*, pages 55–60.

McCann, L. D., McCuller, M. I., Carlton, J. T., Keith, I., Geller, J. B., and Ruiz, G. M. (2019). Bryozoa (Cheilostomata, Ctenostomata, and Cyclostomata) in Galapagos Island fouling communities. *Aquatic Invasions*, 14(1):85–131.

Nakatani, S. (2010). Language detection library for java. https://github.com/shuyo/language-detection.

Patterson, T. (2019). Free vector and raster map data. http://www.naturalearthdata.com/. Accessed: 2019-11-27.

Pedregosa, F., Varoquaux, G., Gramfort, A., Michel, V., Thirion, B., Grisel, O., Blondel, M., Prettenhofer, P., Weiss, R., Dubourg, V., et al. (2011). Scikit-learn: Machine learning in python. *the Journal of machine Learning research*, 12:2825–2830.

Peters, M. E., Neumann, M., Iyyer, M., Gardner, M., Clark, C., Lee, K., and Zettlemoyer, L. (2018a). Deep contextualized word representations. In *Proceedings of the 2018 Conference of the North American Chapter of the Association for Computational Linguistics: Human Language Technologies, Volume 1 (Long Papers)*, pages 2227–2237, New Orleans, Louisiana. Association for Computational Linguistics.

Peters, S. E., Husson, J. M., and Czaplewski, J. (2018b). Macrostrat: a platform for geological data integration and deep-time earth crust research. *Geochemistry, Geophysics, Geosystems*, 19(4):1393–1409.

Provoost, P. and Bosch, S. (2021). *robis: Ocean Biodiversity Information System (OBIS) Client*. R package version 2.8.2.

Rohde, R. (2022). Geowhen. http://www.stratigraphy.org/bak/geowhen/index.html. Accessed: 2022-02-28.

Rumelhart, D. E., Hinton, G. E., and Williams, R. J. (1986). Learning representations by back-propagating errors. *Nature*, 323(6088):533–536.

Salah, M. and Boxer, B. (2022). Mediterranean sea. encyclopedia britannica. http://www.bryozoa.net. Accessed: 2022-21-02.

Schlichtkrull, M., Kipf, T. N., Bloem, P., van den Berg, R., Titov, I., and Welling, M. (2018). Modeling relational data with graph convolutional networks. In *European semantic web conference*, pages 593–607. Springer.

Tilbrook, K. J., Hayward, P., and Gordon, D. (2001). Cheilostomatous Bryozoa from Vanuatu. *Zoological Journal of the Linnean Society*, 131(1):35–109.

WoRMS Editorial Board (2022). World register of marine species (worms). https://www.marinespecies.org. Accessed: 2022-03-14.
