# Supplementary material for: Enhancing georeferenced biodiversity inventories: automated information extraction from literature records reveal the gaps
Source: PeerJ. 2022 Aug 18;10:e13921. doi: 10.7717/peerj.13921 (PMC9393005; doi:10.7717/peerj.13921)
Supplement: Supplemental Information 2 [file peerj-10-13921-s002.pdf]

Appendix S2: TMO References

Abbott, M. B. (1973). Seasonal diversity and density in bryozoan populations of Block Island Sound (New York, U.S.A.). In G. P. Larwood (Ed.), *Living and Fossil Bryozoa* (pp. 37–51). London: Academic Press.

Abdelsalam, K. M. (2014). Benthic bryozoan fauna from the northern Egyptian coast. *Egyptian Journal of Aquatic Research*, *40*, 269–282.

Abdelsalam, K. M. (2016a). Fouling bryozoan fauna from Hurghada, Red Sea, Egypt. I. Erect species. *International Journal of Environmental Science and Engineering*, *7*, 59–70.

Abdelsalam, K. M. (2016b). Fouling bryozoan fauna from Hurghada, Red Sea, Egypt. II. Encrusting species. *Egyptian Journal of Aquatic Research*, *42*, 427–436.

Abdelsalam, K. M., & Ramadan, S. E. (2008a). Fouling Bryozoa from some Alexandria harbours, Egypt. (I) Erect species. *Mediterranean Marine Science*, *9*(1), 31–47.

Abdelsalam, K. M., & Ramadan, S. E. (2008b). Fouling Bryozoa from some Alexandria harbours, Egypt. (II) Encrusting species. *Mediterranean Marine Science*, *9*(2), 5–20.

Abdelsalam, K. M., Taylor, P. D., & Dorgham, M. M. (2017). A new species of *Calyptotheca* (Bryozoa: Cheilostomata) from Alexandria, Egypt, southeastern Mediterranean. *Zootaxa*, *4276*(4), 582–590.

Alder, J. (1864). Descriptions of new British Polyzoa, with remarks on some imperfectly known species. *Quarterly Journal of Microscopical Science*, *4*, 95–109.

Almeida, A. C. S., Alves, O., Peso-Aguiar, M., Dominguez, J., & Souza, F. (2015). Gymnolaemata bryozoans of Bahia State, Brazil. *Marine Biodiversity Records*, *8*.

Almeida, A. C., & Souza, F. B. (2014). Two new species of cheilostome bryozoans from the South Atlantic Ocean. *Zootaxa*, *3753*(3), 283–290.

Almeida, A. C., Souza, F. B., & Vieira, L. M. (2017). Malacostegine bryozoans (Bryozoa: Cheilostomata) from Bahia State, northeast Brazil: Taxonomy and non-indigenous species. *Marine Biodiversity*, 1–26.

Almeida, A. C., Souza, F., Farias, J., Alves, F. M. A., & Vieira, L. M. (2018). Bryozoa on disarticulated bivalve shells from Todos os Santos Bay, northeastern Brazil, with the description of two new species. *Zootaxa*, *4434*(3), 401–428.

Almeida, A. C. S., & Souza, F. B. C. (2014). Two new species of cheilostome bryozoans from the South Atlantic Ocean. *Zootaxa*, *3753*(3), 283–290.

Almeida, A. C. S., Souza, F. B. C., Gordon, D. P., & Vieira, L. M. (2015a). The non-indigenous bryozoan *Triphyllozoon* (Cheilostomata: Phidoloporidae) in the Atlantic: Morphology and dispersion on the Brazilian coast. *Zoologia*, *32*(6), 476–484.

Almeida, A. C. S., Souza, F. B. C., Menegola, C. M. S., Sanner, J., & Vieira, L. M. (2014). Taxonomic review of the family Colatooeciidae Winston, 2005 (Bryozoa, Cheilostomata), with description of seven new species. *Zootaxa*, *3868*(1), 1–61.

Almeida, A. C. S., Souza, F. B. C., Menegola, C. M. S., & Vieira, L. M. (2017). Diversity of marine bryozoans inhabiting demosponges in northeastern Brazil. *Zootaxa*, *4290*(2), 281–323.

Almeida, A. C. S., Souza, F. B. C., Sanner, J., & Vieira, L. M. (2015b). Taxonomy of recent Adeonidae (Bryozoa, Cheilostomata) from Brazil, with the description of four new species. *Zootaxa*, *4013*(3), 348–368.

Almeida, A., Souza, F. B., & Vieira, L. M. (2018). A new species of *Cellaria* (Bryozoa: Cheilostomata) from northeastern Brazil, with a tabular identification key to the Atlantic species. *Zoologia (Curitiba)*, *35*.

Alves, D. F., Barros-Alves, S. P., Lima, D. J., Cobo, V. J., & Negreiros-Fransozo, M. L. (2013). Brachyuran and anomuran crabs associated with *Schizoporella unicornis* (Ectoprocta, Cheilostomata) from southeastern Brazil. *Anais Da Academia Brasileira de Ciências*, *85*(1), 245–256.

Amui, A.-M., & Kaselowsky, J. (2006). Bryozoa from the Gulf of Aden and the Red Sea. Part I: Collections from the fifth expedition of the R.V. Meteor. *Fauna of Arabia*, *22*, 7–22.

Anderson, C. M., & Haygood, M. G. (2007). A-Proteobacterial symbionts of marine bryozoans in the genus *Watersipora*. *Applied and Environmental Microbiology*, *73*(1), 303–311.

Arístegui, J. (1985). The genus *Adeonellopsis* MacGillivray (Bryozoa: Cheilostomata) in the Canary Islands: *A. Distoma* (Busk) and *A. Multiporosa* sp. Nov. *Journal of Natural History*, *19*, 425–430.

Ayari, R., & Taylor, P. D. (2014). Some bryozoans from Tunisia, Western Mediterranean Sea. *Studi Trentini Di Scienze Naturali*, *94*, 11–20.

Álvarez, J. A. (1990). Notes on two species of the genus *Turbicellepora* Ryland, 1963 (Bryozoa, Cheilostomatida) of the Atlanto-Mediterranean region: *T. avicularis* (Hincks, 1860) and *t. Magnicostata* (Barroso, 1919). *Cahiers de Biologie Marine*, *31*, 473–483.

Bader, B., & Schäfer, P. (2005). Bryozoans in polar latitudes: Arctic and Antarctic bryozoan communities and facies. *Denisia*, *16*, 263–282.

Balazy, P., & Kuklinski, P. (2013). Mobile hard substrata–An additional biodiversity source in a high latitude shallow subtidal system. *Estuarine, Coastal and Shelf Science*, *119*, 153–161.

Banta, W. C. (1969). The recent introduction of *watersipora arcuata* Banta (Bryozoa, Cheilostomata) as a fouling pest in southern California. *Bulletin of the Southern California Academy of Sciences*, *68*(4), 248–251.

Banta, W. C. (1969). *Watersipora arcuata*, a new species in the subovoidea - cucullata - nigra complex (Bryozoa, Cheilostomata). *Bulletin of the Southern California Academy of Sciences*, *68*, 96–102.

Banta, W. C. (1991). The Bryozoa of the Galapagos. In M. James (Ed.), *Galapagos marine invertebrates: Taxonomy, biogeography and evolution in Darwin's islands* (pp. 371–389). New York: Plenum Publishing Company.

Banta, W. C., Perez, F. M., & Santagata, S. (1995). A setigerous collar in Membranipora chesapeakensis n. Sp.(Bryozoa): Implications for the evolution of cheilostomes from ctenostomes. *Invertebrate Biology*, 83–88. Retrieved from http://www.jstor.org/stable/10.2307/3226957

Banta, W. C., & Santagata, S. (1996). Annotated species list of the Bryozoa of Chuuk, Federated States of Micronesia. *Micronesica*, *29*(2), 305–312.

Barnes, D. K. A. (2006). A most isolated benthos: Coastal bryozoans of Bouvet Island. *Polar Biology*, *29*, 114–119.

Barnes, D. K. A., & Griffiths, H. J. (2008). Biodiversity and biogeography of southern temperate and polar bryozoans. *Global Ecology and Biogeography*, *17*, 84–99.

Barnes, D. K. A., & Kuklinski, P. (2004). Scale-dependent variation in competitive ability among encrusting Arctic species. *Marine Ecology Progress Series*, *275*, 21–32.

Barnes, D. K. A., & Kuklinski, P. (2010). Bryozoans of the Weddell Sea continental shelf, slope and abyss: Did marine life colonize the Antarctic shelf from deep water, outlying islands or in situ refugia following glaciations? *Journal of Biogeography*, *37*(9), 1648–1656.

Barnes, D. K. A., Kuklinski, P., Jackson, J. A., Keel, G. W., Morley, S. A., & Winston, J. E. (2011). Scott's collections help reveal accelerating marine life growth in Antarctica. *Current Biology*, *21*(4), R147–R148. https://doi.org/10.1016/j.cub.2011.01.033

Barnes, D. K. A., Webb, K. E., & Linse, K. (2007). Growth rate and its variability in erect Antarctic bryozoans. *Polar Biology*, *30*, 1069–1081.

Barnes, D. K., & Clarke, A. (1998). The ecology of an assemblage dominant: The encrusting bryozoan *Fenestrulina rugula*. *Invertebrate Biology*, *117*(4), 331–340.

Bastos, A. C., Moura, R. L., Moraes, F. C., Vieira, L. S., Braga, J. C., Ramalho, L. V., … Webster, J. M. (2018). Bryozoans are Major Modern Builders of South Atlantic Oddly Shaped Reefs. *Scientific Reports*, *8*(1), 9638.

Bayer, M. M., & Todd, C. D. (1997). Evidence for zooid senescence in the marine bryozoan *electra pilosa*. *Invertebrate Biology*, *116*(4), 331–340.

Bayer, M. M., Todd, C. D., Hoyle, J. E., & Wilson, J. F. B. (1997). Wave-related abrasion induces formation of extended spines in a marine bryozoan. *Proceedings of the Royal Society of London B*, *264*, 1605–1611.

Beaver, M., & von Dassow, M. (2013). *Feeding the Masses: Colonial Transport in the Marine Bryozoan Membranipora membranacea.* Retrieved from https://digital.lib.washington.edu/researchworks/handle/1773/27199

Benedix, G., Jacob, D. E., & Taylor, P. D. (2014). Bimineralic bryozoan skeletons: A comparison of three modern genera. *Facies*, *60*(2), 389–403. https://doi.org/10.1007/s10347-013-0380-2

Berning, B. (2012). Taxonomic notes on some Cheilostomata (Bryozoa) from Madeira. *Zootaxa*, *3236*, 36–54.

Berning, B. (2013). New and little-known Cheilostomata (Bryozoa, Gymnolaemata) from the NE Atlantic. *European Journal of Taxonomy*, *44*, 1–25. https://doi.org/10.5852/ejt.2013.44

Berning, B., Achilleos, K., & Wisshak, M. (2019). Revision of the type species of *Metroperiella* (Bryozoa, Cheilostomatida), with the description of two new species. *Journal of Natural History*, *53*(3-4), 141–158. https://doi.org/10.1080/00222933.2019.1582725

Berning, B., Harmelin, J.-G., & Bader, B. (2017). New Cheilostomata (Bryozoa) from NE Atlantic seamounts, islands, and the continental slope: Evidence for deep-sea endemism. *European Journal of Taxonomy*, *347*, 1–51. https://doi.org/10.5852/ejt.2017.347

Berning, B., & Kuklinski, P. (2008). North-east Atlantic and Mediterranean species of the genus *Buffonellaria* (Bryozoa, Cheilostomata): Implications for biodiversity and biogeography. *Zoological Journal of the Linnean Society*, *152*(3), 537–566. https://doi.org/10.1111/j.1096-3642.2007.00379.x

Berning, B., & Ostrovsky, A. N. (2011). *Omanipora pilleri* nov. Gen. Nov. Spec., a new lepraliomorph bryozoan (Cheilostomata) from Oman. *Annalen Des Naturhistorischen Museums in Wien*, *113*, 511–523.

Berning, B., Tilbrook, K. J., & Ostrovsky, A. N. (2014). What, if anything, is a lyrula? *Studi Trentini Di Scienze Naturali*, *94*, 21–28.

Beukhof, E. D., Coolen, J. W. P., van der Weide, B. E., Cuperus, J., de Blauwe, H., & Lust, J. (2016). Records of five bryozoan species from offshore gas platforms rare for the Dutch North Sea. *Marine Biodiversity Records*, *9*(1). https://doi.org/10.1186/s41200-016-0086-6

Bishop, J. D. D. (1989). Colony form and the exploitation of spatial refuges by encrusting Bryozoa. *Biological Reviews*, *64*, 197–218.

Bock, P. E., & Cook, P. L. (2004a). Dimorphic brooding zooids in the genus *Adeona* Lamouroux from Australia (Bryozoa: Cheilostomata). *Memoirs of Museum Victoria*, *61*(2), 129–133.

Bock, P. E., & Cook, P. L. (2004b). New species of the bryozoan genera *Batopora* and Lacrimula (Batoporidae) from Australia. *Proceedings of the Royal Society of Victoria*, *116*(2), 283–288.

Bone, E. K., & Keough, M. J. (2005). Responses to damage in an arborescent bryozoan: Effects of injury location. *Journal of Experimental Marine Biology and Ecology*, *324*(2), 127–140.

Bone, E. K., & Keough, M. J. (2010). Does Polymorphism Predict Physiological Connectedness? A Test Using Two Encrusting Bryozoans. *The Biological Bulletin*, *219*(3), 220–230.

Boonzaaier, M. K., Florence, W. K., & Jones, M. S. (2014). Historical review of South African bryozoology: A legacy of European endeavour. *Annals of Bryozoology 4*, *4*.

Borg, F. (1930). On the bryozoan Fauna of Skelderviken. *Arkiv För Zoologi*, *21 A*(24), 1–13.

Bowden, D. A. (2005a). Quantitative characterization of shallow marine benthic assemblages at Ryder Bay, Adelaide Island, Antarctica. *Marine Biology*, *146*, 1235–1249.

Bowden, D. A. (2005b). Seasonality of recruitment in Antarctic sessile marine benthos. *Marine Ecology Progress Series*, *297*, 101–118.

Bradstock, M., & Gordon, D. P. (1983). Coral-like bryozoan growths in Tasman Bay, and their protection to conserve commercial fish stocks. *New Zealand Journal of Marine and Freshwater Research*, *17*, 159–163.

Branch, M. L., & Hayward, P. (2005). New species of cheilostomatous Bryozoa from subantarctic Marion and Prince Edward Islands. *Journal of Natural History*, *39*(29), 2671–2704.

Branch, M. L., & Hayward, P. J. (2007). The Bryozoa of subantarctic Marion and Prince Edward Islands: Illustrated keys to the species and results of the 1982-1989 University of Cape Town surveys. *African Journal of Marine Science*, *29*(1), 1–24.

Brown, D. A. (1949). On the polyzoan genus *Hippomenella* Canu & Bassler and its genotype Lepralia mucronelliformis Waters. *Journal of the Linnean Society, London (Zoology)*, *41*, 513–520.

Burgess, S. C., & Marshall, D. J. (2011). Temperature-induced maternal effects and environmental predictability. *Journal of Experimental Biology*, *214*(14), 2329–2336. https://doi.org/10.1242/jeb.054718

Busk, G. (1852). *Catalogue of Marine Polyzoa in the Collection of the British Museum. Part II. Cheilostomata (Part).* London: Trustees of the British Museum.

Busk, G. (1875). *Catalogue of marine Polyzoa in the collection of the British Museum. Part III. Cyclostomata.* London: Trustees of the British Museum.

Cadée, G. C., Chimonides, P. J., & Cook, P. L. (1989). *Pseudolunularia* gen.n. (Cheilostomata), a lunulitiform bryozoan from the Indo-West Pacific. *Zoologica Scripta*, *18*(1), 43–48.

Cancino, J. M., Hughes, R. N., & Orellana, M. C. (1994). *A comparative study of larval release in bryozoans* (P. J. Hayward, Ed.). Olsen & Olsen, Fredensborg.

Canning-Clode, J., Souto, J., & McCann, L. (2013). First record of textitCelleporaria brunnea (Bryozoa: Lepraliellidae) in Portugal and in the East Atlantic. *Marine Biodiversity Records*, *6*, 1–5.

Cardigos, F., Tempera, F., Ávila, S. P., Gonçalves, J., Colaço, A., & Santos, R. S. (2006). Non-indigenous marine species of the Azores. *Helgoland Marine Research*, *60*, 160–169.

Carlton, J. T., Chapman, J. W., Geller, J. B., Miller, J. A., Ruiz, G. M., Carlton, D. A., ... Breitenstein, R. A. (2018). Ecological and biological studies of ocean rafting: Japanese tsunami marine debris in North America and the Hawaiian Islands. *Aquatic Invasions*, *13*(1), 1–9.

Carter, M. C., & Gordon, D. P. (2007). Substratum and morphometric relationships in the bryozoan genus *Odontoporella*, with a description of a new paguridean-symbiont species from New Zealand. *Zoological Science*, *24*, 47–56.

Centurión, R., & López Gappa, J. (2011). Bryozoan assemblages on hard substrata: Species abundance distribution and competition for space. *Hydrobiologia*, *658*, 329–341. https://doi.org/10.1007/s10750-010-0503-5

César-Aldariz, J., Fernández Pulpeiro, E., & Reverter Gil, O. (1999). A new species of the genus *Celleporella* (Broyzoa: Cheilostomatida) from the European Atlantic coast. *Journal of the Marine Biological Association*, *79*(1), 51–55.

Chainho, P., Fernandes, A., Amorim, A., Ávila, S. P., Canning-Clode, J., Castro, J. J., … Costa, M. J. (2015). Non-indigenous species in Portuguese coastal areas, coastal lagoons, estuaries and islands. *Estuarine, Coastal and Shelf Science*, *167*, 199–211.

Chandramouli, K. H., Zhang, Y., Wong, Y. H., & Qian, P.-Y. (2011). Comparative glycoproteome analysis: Dynamics of protein glycosylation during metamorphic transition from pelagic to benthic life stages in three invertebrates. *Journal of Proteome Research*, *11*(2), 1330–1340.

Cheetham, A. H. (2014). Jeremy Jackson, Bryozoa, and the Punctuated Equilibrium Debate. In S. J. Wyse Jackson Patrick N. (Ed.), *Annals of Bryozoology 4: Aspects of the history of research on bryozoans.* (Vol. 4, pp. 35–52). Dublin: International Bryozoology Association.

Cheetham, A. H., & Hayek, L.-A. C. (1988). Phylogeny reconstruction in the Neogene bryozoan *Metrarabdotos*: A paleontologic evaluation of methodology. *Historical Biology*, *1*(1), 65–83.

Chimenz, C., Nicoletti, L., & Lippi Boncambi, F. (1997). First record of the genus *Retevirgula* in the Mediterranean Sea, with description of R. *Akdenizae* sp. N. (Bryozoa, Cheilostomatida). *Italian Journal of Zoology*, *64*(3), 279–282.

Chimenz Gusso, C., & Tomassetti, P. (2004). Bryozoa Gymnolaemata from the coast of Tunisia and the Strait of Sicily. *Biologia Marina Mediterranea*, *11*(2), 412–415.

Chimonides, P. J., & Cook, P. L. (1994). Notes on the genus *Cranosina* (Bryozoa, Cheilostomida). *Zoologia Scripta*, *23*(1), 43–49.

Chiriboga, A., Ruiz, D., & Banks, S. (2012). CDF Checklist of Galapagos Bryozoans. In *Charles Darwin Foundation Galapagos species checklist.*

Cocito, S., & Ferdeghini, F. (2001). Carbonate standing stock and carbonate production of the bryozoan Pentapora fascialis in the north-western Mediterranean. *Facies*, *45*, 25–30.

Conroy, T. J., Cook, P. L., & Bock, P. E. (2001). New species of *Otionellina* and *Selenaria* (Bryozoa-Cheilostomata) from the South West Shelf, Western Australia. *Transactions of the Royal Society of South Australia*, *125*(1), 15–23.

Cook, E. J., Stehlíková, J., Beveridge, C. M., Burrows, M. T., De Blauwe, H., & Faasse, M. (2013). Distribution of the invasive bryozoan *Tricellaria inopinata* in Scotland and a review of its European expansion. *Aquatic Invasions*, *8*(3).

Cook, P. L. (1966). Some "sand fauna" Polyzoa (Bryozoa) from eastern Africa and the northern Indian Ocean. *Cahiers de Biologie Marine*, *7*, 207–223.

Cook, P. L., & Bock, P. E. (1994). The astogeny and morphology of *Rhabdozoum wilsoni* Hincks (Anasca, Buguloidea). In P. J. Hayward, J. S. Ryland, & P. D. Taylor (Eds.), *Biology and Palaeobiology of Bryozoans* (pp. 47–50). Fredensborg: Olsen & Olsen.

Cook, P. L., & Bock, P. E. (2000). Two new genera of Bryozoa (Calloporidae) from New Zealand. *Journal of Natural History*, *34*(7), 1125–1133.

Cook, P. L., & Hayward, P. J. (1983). Notes on the family Lekythoporidae (Bryozoa, Cheilostomata). *Bulletin of the British Museum (Natural History), Zoology*, *45*(2), 55–76.

Corriero, G., Longo, C., Mercurio, M., Marchini, A., & Occhipinti-Ambrogi, A. (2007). Porifera and Bryozoa on artificial hard bottoms in the Venice Lagoon: Spatial distribution and temporal changes in the northern basin. *Italian Journal of Zoology*, *74*(1), 21–29. https://doi.org/10.1080/11250000601084100

Cortés, J., Nielsen, V., & Herrera-Cubilla, A. (2009). Bryozoans. In I. S. Wehrtmann & J. Cortés (Eds.), *Marine Biodiversity of Costa Rica* (Vol. 86, pp. 385–388, 413–416).

Cranfield, H. J., Gordon, D. P., Willan, R. C., Marshall, B. A., Battershill, C. N., Francis, M. P., ... Read, G. B. (1998). Adventive marine species in New Zealand. *NIWA Technical Report*, *34*, 1–48.

Cranfield, H. J., Rowden, A. A., Smith, D. J., Gordon, D. P., & Michael, K. P. (2004). Macrofaunal assemblages of benthic habitat of different complexity and the proposition of a model of biogenic reef habitat regeneration in Foveaux Strait, New Zealand. *Journal of Sea Research*, *52*, 109–125.

Cukrov, M., & Novosel, M. (2014). New records of the estuarine bryozoan *Conopeum seurati* (Canu, 1928) along the eastern (Croatian) coast of the Adriatic Sea. *Studi Trentini Di Scienze Naturali*, *94*, 39–44.

Cumming, R. L. (2015). Two tropical species of *Stephanotheca* (Bryozoa, Cheilostomata, Lanceoporidae) from the Gulf of Carpentaria, Australia. *Zootaxa*, *3948*(2), 279–286.

Cumming, R. L., & Sebastian, P. (2018). New encrusting species of Lanceoporidae (Bryozoa, Cheilostomata) from the southern Great Barrier Reef, Australia. *Zootaxa*, *4500*(1), 104. https://doi.org/10.11646/zootaxa.4500.1.6

Cumming, R. L., & Tilbrook, K. J. (2014). Six species of *Calyptotheca* (Bryozoa, Cheilostomata, Lanceoporidae) from the Gulf of Carpentaria and northern Australia, with description of a new species. *Zootaxa*, *3827*(2), 147–169.

De Blauwe, H. (2005). A new species of Caulibugula (Bryozoa: Cheilostomatida) from France. *Bulletin de L'Institut Royal Des Sciences Naturelles de Belgique, Biologie*, *75*, 81–87.

De Blauwe, H. (2006). On the taxonomy and distribution of the family Pacificincolidae Liu & Liu, 1999 (Bryozoa, Cheilostomata), with the description of a new genus. *Bulletin de L'Institut Royal Des Sciences Naturelles de Belgique, Biologie*, *76*, 139–145.

De Blauwe, H., & Faasse, M. (2001). Extension of the range of the bryozoans Tricellaria inopinata and Bugula simplex in the North-East Atlantic Ocean (Bryozoa: Cheilostomatida). *Nederlandse Faunistische Mededelingen*, *14*, 103–112.

De Blauwe, H., & Faasse, M. (2004). Smittoidea prolifica Osburn, 1952 (Bryozoa, Cheilostomatida), a Pacific bryozoan introduced to The Netherlands (Northeast Atlantic). *Bulletin de L'Institut Royal Des Sciences Naturelles de Belgique, Biologie*, *74*, 33–39.

De Blauwe, H., & Gordon, D. P. (2014). New bryozoan taxa from a biodiversity hotspot in the Eastern Weddell Sea. *Studi Trentini Di Scienze Naturali*, *94*, 53–78.

De Blauwe, H., Kind, B., Kuhlenkamp, R., Cuperus, J., van der Weide, B., & Kerckhof, F. (2014). Recent observations of the introduced *Fenestrulina Delicia* Winston, Hayward & Craig, 2000 (Bryozoa) in Western Europe. *Studi Trentini Di Scienze Naturali*, *94*, 45–51.

de la Nuez-Hernández, D., Valle, C., Forcada, A., Gonzálz-Correa, J. M., & Fernández Torquemada, Y. (2014). Assessing the erect bryozoan *Myriapora truncata* (Pallas, 1766) as indicator of recreational diving impact on coralligenous reef communities. *Ecological Indicators*, *46*, 193–200.

Denisenko, N. V. (2011). Bryozoans of the East Siberian Sea: History of research and current knowledge of diversity. In *3rd Ser. Annals of Bryozoology 3: Aspects of the history of research on bryozoans* (pp. 1–15).

Denisenko, N. V. (2014). Deep-sea fauna of European seas: An annotated species checklist of benthic invertebrates living deeper than 2000 m in the seas bordering Europe. Bryozoa. *Invertebrate Zoology*, *11*(1), 89–98.

Denisenko, N. V. (2015). New species of the genus *Parasmittina* (Bryozoa: Cheilostomata: Smittinidae) from the Chukchi Sea. *Zoosystemica Rossica*, *24*(2), 303–306.

Denisenko, N. V. (2016). A new species of the genus *Callopora* (Bryozoa: Cheilostomatida: Calloporidae) from the Barents and Kara Seas. *Zoosystematica Rossica*, *25*(2), 183–188.

Denisenko, N. V. (2016). Two new species of the genus *Turbicellepora* Ryland, 1963 (Bryozoa: Celleporidae) found on Lophelia coral from the Greenland slope. *Zootaxa*, *4066*(2), 177–182.

Denisenko, N. V. (2018). New Cheilostomata (Bryozoa) species from sublittoral and bathyal zones off the Faroe Islands, with some comments on allied taxa. *Zootaxa*, *4375*(1), 116–126.

Denisenko, N. V., & Kukliński, P. (2008). Historical development of research and current state of bryozoan diversity in the Chukchi Sea. In P. N. Wyse Jackson & S. Jones (Eds.), *Annals of Bryozoology 2: Aspects of the history of research on bryozoans.* (pp. 35–50). Dublin: International Bryozoology Association.

Denisenko, N. V., Thomsen, E., & Tendal, O. S. (2013). Bryozoan epifauna on brachiopods from the Faroe Islands (NE Atlantic). *Fró skaparrit*, *60*, 96–113.

d'Hondt, J.-L. (2002). The French Pre-Lamarckian bryozoologists. In P. N. Wyse Jackson & M. E. Spencer Jones (Eds.), *Annals of Bryozoology: Aspects of the history of research on bryozoans* (pp. 81–95). Dublin: International Bryozoology Association.

d'Hondt, J.-L. (2008). The historical collections of Recent Bryozoa in the French National Collections. In P. N. Wyse Jackson & M. E. Spencer Jones (Eds.), *Annals of Bryozoology 2: Aspects of the history of research on bryozoans.* (pp. 59–70). Dublin: International Bryozoology Association.

d'Hondt, J.-L., & Mascarell, G. (2004). On some Bryozoa collected during the northern cruises of the "Pourquoi-Pas" during the years 1921-1929 and description of a new species. *Cahiers de Biologie Marine*, *45*, 281–285.

Dick, M. H. (2008). Unexpectedly high diversity of *Monoporella* (Bryozoa: Cheilostomata) in the Aleutian Islands, Alaska: Taxonomy and distribution of six new species. *Zoological Science*, *25*, 36–52.

Dick, M. H., & Grischenko, A. V. (2017). Rocky-intertidal cheilostome bryozoans from the vicinity of the Sesoko Biological Station, west-central Okinawa, Japan. *Journal of Natural History*, *51*(3-4), 141–266.

Dick, M. H., Grischenko, A. V., & Mawatari, S. F. (2005). Intertidal Bryozoa (Cheilostomata) of Ketchikan, Alaska. *Journal of Natural History*, *39*(43), 3687–3784.

Dick, M. H., Mawatari, S. F., Sanner, J., & Grischenko, A. V. (2011). Cribrimorph and Other Cauloramphus Species (Bryozoa: Cheilostomata) from the Northwestern Pacific. *Zoological Science*, *28*(2), 134–147. https://doi.org/10.2108/ZSJ.28.134

Dick, M. H., Tilbrook, K. J., & Mawatari, S. F. (2006). Diversity and taxonomy of rocky-intertidal Bryozoa on the Island of Hawaii, USA. *Journal of Natural History*, *40*(38-40), 2197–2257.

D'Onghia, G., Cape, F., uto, Cardone, F., Carlucci, R., Carluccio, A., Chimienti, G., … Tursi, A. (2015). Macro- and megafauna recorded in the submarine Bari Canyon (southern Adriatic, Mediterranean Sea) using different tools. *Mediterranean Marine Science*, *16*(1), 180–196.

Dumont, J. P. C. (1981). A report on the cheilostome Bryozoa of the Sudanese Red Sea. *Journal of Natural History*, *15*, 623–637.

Durrant, H. M. S., Clark, G. F., Dworjanyn, S. A., Byrne, M., & Johnston, E. L. (2013). Seasonal variation in the effects of ocean warming and acidification on a native bryozoan, *Celleporaria nodulosa. Marine Biology*, *2013*(8), 1903–1911. https://doi.org/DOI%2010.1007/s00227-012-2008-4

Dyrynda, P. E. J., & Ryland, J. S. (1982). Reproductive strategies and life histories in the cheilostome marine bryozoans *Chartella papyracea* and *Bugula* flabellata. *Marine Biology*, *71*, 241–256.

Faasse, M., Van Moorsel, G., & Tempelman, D. (2013). Moss animals of the Dutch part of the North Sea and coastal waters of the Netherlands (Bryozoa). *Nederlandse Faunistische Mededelingen*, *41*, 1–14.

Fehlauer-Ale, K. H., Mackie, J. A., Lim-Fong, G. E., Ale, E., Pie, M. R., & Waeschenbach, A. (2014). Cryptic species in the cosmopolitan *Bugula neritina* complex (Bryozoa, Cheilostomata). *Zoologica Scripta*, *43*(2), 193–205.

Fehlauer-Ale, K. H., Winston, J. E., Tilbrook, K. J., Nascimento, K. B., & Vieira, L. M. (2015). Identifying monophyletic groups within Bugula sensu lato (Bryozoa, Buguloidea). *Zoologica Scripta*.

Ferrario, J., d'Hondt, J.-L., Marchini, A., & Occhipinti-Ambrogi, A. (2015). From the Pacific Ocean to the Mediterranean Sea: Watersipora arcuata, a new non-indigenous bryozoan in Europe. *Marine Biology Research*, *11*(9), 909–919.

Ferrario, J., Rosso, A., Marchini, A., & Occhipinti-Ambrogi, A. (2018). Mediterranean non-indigenous bryozoans: An update and knowledge gaps. *Biodiversity and Conservation*, *27*(11), 2783–2794. https://doi.org/10.1007/s10531-018-1566-2

Ferretti, C., Magnino, G., & Balduzzi, A. (2007). Morphology of the larva and ancestrula of *Myriapora truncata* (Bryozoa, Cheilostomatida). *Italian Journal of Zoology*, *74*(4), 341–350. https://doi.org/10.1080/11250000701629572

Figuerola, B., Angulo-Preckler, C., Núñez-Pons, L., Moles, J., Sala-Comorera, L., García-Aljaro, C., … Avila, C. (2017). Experimental evidence of chemical defence mechanisms in Antarctic bryozoans. *Marine Environmental Research*, *129*, 68–75.

Figuerola, B., Ballesteros, M., & Avila, C. (2013). Description of a new species of Reteporella (Bryozoa: Phidoloporidae) from the Weddell Sea (Antarctica) and the possible functional morphology of avicularia. *Acta Zoologica*, *94*(1), 66–73.

Figuerola, B., Barnes, D. K., Brickle, P., & Brewin, P. E. (2017). Bryozoan diversity around the Falkland and South Georgia Islands: Overcoming Antarctic barriers. *Marine Environmental Research*, *126*, 81–94.

Figuerola, B., Gordon, D. P., & Cristobo, J. (2018). New deep Cheilostomata (Bryozoa) species from the Southwestern Atlantic: Shedding light in the dark. *Zootaxa*, *4375*(2), 211–249. https://doi.org/10.11646/zootaxa.4375.2.3

Figuerola, B., Gore, D. B., Johnstone, G., & Stark, J. S. (2019). Spatio-temporal variation of skeletal Mg-calcite in Antarctic marine calcifiers. *PLOS ONE*, *14*(5). https://doi.org/10.1371/journal.pone.0210231

Figuerola, B., Monleón-Getino, T., Ballesteros, M., & Avila, C. (2012). Spatial patterns and diversity of bryozoan communities from the Southern Ocean: South Shetland Islands, Bouvet Island and Eastern Weddell Sea. *Systematics and Biodiversity*, *10*(1), 109–123.

Figuerola, B., Núñez-Pons, L., Moles, J., & Avila, C. (2013). Feeding repellence in Antarctic bryozoans. *Naturwissenschaften*, *100*, 1069–1081. https://doi.org/10.1007/s00114-013-1112-8

Figuerola, B., Núñez-Pons, L., Monleón-Getino, T., & Avila, C. (2014). Chemo-ecological interactions in Antarctic bryozoans. *Polar Biology*, *37*, 1017–1030.

Figuerola, B., Sala-Comorera, L., Angulo-Preckler, C., Vázquez, J., Montes, M. J., García-Aljaro, C., … Avila, C. (2014). Antimicrobial activity of Antarctic bryozoans: An

ecological perspective with potential for clinical applications. *Marine Environmental Research*, *101*, 52–59.

Figuerola, B., Taboada, S., Monleón-Getino, T., Vázquez, J., & Avila, C. (2013). Cytotoxic activity of Antarctic benthic organisms against the common sea urchin *Sterechinus neumayeri*. *Oceanography*, *1*(107), 2.

Florence, W. K., Hayward, P. J., & Gibbons, M. J. (2007). Taxonomy of shallow-water Bryozoa from the west coast of South Africa. *African Natural History*, *3*, 1–58.

Fortunato, H. (2015). Bryozoans in climate and ocean acidification research: A reappraisal of an under-used tool. *Regional Studies in Marine Science*, *2, Supplement*, 32–44. https://doi.org/10.1016/j.rsma.2015.08.010

Fortunato, H., Roloff, A., & Schäfer, P. (2014). Salinity effects on growth and development of Flustra foliacea (L.) Zooids: Experimental data. *Studi Trentini Di Scienze Naturali*, *94*, 111–118.

Fortunato, H., Schäfer, P., & Blaschek, H. (2012). Growth rates, age determination, and calcification levels in *Flustra foliacea* (L.) (Bryozoa: Cheilostomata): Preliminary assessment. In A. Ernst, P. Schäfer, & J. Scholz (Eds.), *Bryozoan Studies 2010* (pp. 59–74). Berlin: Springer.

Fortunato, H., & Spencer Jones, M. E. (2014). Collections and climate change research: *Flustra foliacea* (L.) (Bryozoa) in the Natural History Museum, London. *International Bryozoology Association, Dublin*, 53–73.

Franjević, D., Novosel, M., & Koletić, N. (2015). Freshwater and brackish bryozoan species of Croatia (Bryozoa: Gymnolaemata, Phylactolaemata) and their genetic identification. *Zootaxa*, *4032*(2), 221–228.

Fransen, C. H. J. M. (1986). Caribbean Bryozoa: Anasca and Ascophora Imperfecta of the inner bays of Curaçao and Bonaire. *Studies on the Fauna of Curaçao and Other Caribbean Islands*, *68*, 1–119.

Fuchs, J., Martindale, M. Q., & Hejnol, A. (2011). Gene expression in bryozoan larvae suggest a fundamental importance of pre-patterned blastemic cells in the bryozoan life-cycle. *EvoDevo*, *2*(1), 1–15.

Førde, H., Forbord, S., Handå, A., Fossberg, J., Arff, J., Johnsen, G., & Reitan, K. I. (2016). Development of bryozoan fouling on cultivated kelp (*Saccharina latissima*) in Norway. *Journal of Applied Phycology*, *28*(2), 1225–1234. https://doi.org/10.1007/s10811-015-0606-5

Gavira-O'Neill, K., Guerra-García, J. M., Moreira, J., & Ros, M. (2018). Mobile epifauna of the invasive bryozoan Tricellaria inopinata: Is there a potential invasional meltdown? *Marine Biodiversity*, *48*(2), 1169–1178. https://doi.org/10.1007/s12526-016-0563-5

Gerovasileiou, V., & Rosso, A. (2016). Marine Bryozoa of Greece: An annotated checklist. *Biodiversity Data Journal*, *4*, e10672. https://doi.org/doi:%2010.3897/BDJ.4.e10672

Gluhak, T., Lewis, J. E., & Popijac, A. (2007). Bryozoan fauna of Green Island, Taiwan: First indications of biodiversity. *Zoological Studies*, *46*(4), 397–426.

Gontar, V. I. (1993). New deepwater species of Cheilostomida from the Kuril Islands and the Pacific Ocean (Bryozoa). *Zoosystematica Rossica*, *2*(1), 41–45.

Gontar, V. I. (2008). Three new species of the genus Smittina from the Weddell Sea, Antarctic (Bryozoa: Cheilostomata: Smittinidae). *Zoosystematica Rossica*, *17*(1), 7–9.

Gontar, V. I. (2012a). Evolution and distribution of the Antarctic and Arcitc cheilostomate Bryozoa. *Ecology & Safety*, *6*(1), 206–211.

Gontar, V. I. (2012b). The fauna of Bryozoa Cheilostomata of the Black Sea. *Ecology & Safety*, *6*(3), 100–129.

Gontar, V. I. (2013). Bryozoa of the Russian sea shore of the Baltic Sea. *Journal of International Scientific Publications: Ecology & Safety*, *7*(3), 99–107.

Gontar, V. I. (2014). Invasive species in the fauna of Bryozoa in the Bering and the Chuckchi Seas. *Journal of International Scientific Publications: Ecology & Safety*, *8*, 354–360.

Gontar, V. I. (2014). New additions to the fauna of Bryozoa Cheilostomata of the Black Sea. *Journal of International Scientific Publications: Ecology and Safety*, *8*, 361–369.

Gontar, V. I. (2017). A new species of the genus Isoschizoporella (Bryozoa: Gymnolaemata: Cheilostomatida: Eminooeciidae) from the Weddell Sea Isoschizoporella (Bryozoa: Gymnolaemata: Cheilostomatida: Eminooeciidae) . *ZOOSYSTEMATICA ROSSICA*, *26*(2), 209–211.

Gordon, D. P. (1971). Zooidal budding in the cheilostomatous Bryozoan Fenestrulina malusii var. Thyreophora. *New Zealand Journal of Marine and Freshwater Research*, *5*, 453–460.

Gordon, D. P. (1972). Biological relationships of an intertidal bryozoan population. *Journal of Natural History*, *6*, 503–514.

Gordon, D. P. (1974). Microarchitecture and function of the lophophore in the bryozoan *Cryptosula pallasiana*. *Marine Biology*, *27*(2), 147–163. https://doi.org/10.1007/BF00389068

Gordon, D. P. (1985). Additional species and records of gymnolaemate Bryozoa from the Kermadec region. *New Zealand Oceanographic Institute Records*, *4*(14), 159–183.

Gordon, D. P. (1988). The bryozoan families Sclerodomidae, Bifaxariidae, and Urceoliporidae and a novel type of frontal wall. *New Zealand Journal of Zoology*, *15*, 249–290.

Gordon, D. P. (1989a). Intertidal bryozoans from coral reef-flat rubble, Sa'aga, Western Samoa. *New Zealand Journal of Zoology*, *16*, 447–463.

Gordon, D. P. (1989b). New and little-known deep-sea taxa of umbonulomorph Bryozoa and their classification. *New Zealand Journal of Zoology*, *16*, 251–267.

Gordon, D. P. (1993). Bryozoa: The ascophorine infraorders Cribriomorpha, Hippothoomorpha and Umbonulomorpha mainly from New Caledonian waters. *Mémoires Du Muséum National d'Histoire Naturelle*, *158*, 299–347.

Gordon, D. P. (2009a). *Baudina* gen. Nov., constituting the first record of Pasytheidae from Australia, and Sinoflustridae fam. Nov., with a checklist of Bryozoa and Pterobranchia from Beagle Gulf. *The Beagle, Records of the Museums and Art Galleries of the Northern Territory*, *25*, 43–54.

Gordon, D. P. (2009b). New bryozoan taxa from a new marine conservation area in New Zealand, with a checklist of Bryozoa from Greater Cook Strait. *Zootaxa*, *1987*, 39–60.

Gordon, D. P. (2016). Bryozoa of the South China Sea - an overview. *Raffles Bulletin of Zoology*, *34*((Supplement)), 604–618.

Gordon, D. P., & Arnold, P. W. (1998). *Bryorachis* (Phidoloporidae) and *Retelepralia* (Cheiloporinidae): Two new genera of Indo-Pacific Bryozoa. *Memoirs of the Queensland Museum*, *42*(2), 495–503.

Gordon, D. P., & Grischenko, A. V. (1994). Bryozoan frontal shields: The types species of Desmacystis, Ramphostomella, Rhamphosmittina, Rhamphostomellina, and new genus Arctonula. *Zoologia Scripta*, *23*(1), 61–72.

Gordon, D. P., & Hastings, A. B. (1979). The interzooidal communications of *Hippothoa* sensu lato (Bryozoa) and their value in classification. *Journal of Natural History*, *13*, 561–579.

Gordon, D. P., & Mawatari, S. F. (1992). Atlas of marine-fouling Bryozoa of New Zealand ports and harbours. *Miscellaneous Publications New Zealand Oceanographic Institute*, *107*, 1–52.

Gordon, D. P., Mawatari, S. F., & Kajihara, H. (2002). New taxa of Japanese and New Zealand Eurystomellidae (Phylum Bryozoa) and their phylogenetic relationships. *Zoological Journal of the Linnean Society*, *136*, 199–216.

Gordon, D. P., & Parker, S. A. (1991). An aberrant new genus and subfamily of the spiculate bryozoan family Thalamoporellidae epiphytic on *Posidonia. Journal of Natural History*, *25*(5), 1363–1378. https://doi.org/10.1080/00222939100770851

Gordon, D. P., & Parker, S. A. (1991). The plectriform apparatus - an enigmatic structure in malacostegine Bryozoa. In F. P. Bigey (Ed.), *Bryozoaires Actuels et Fossiles: Bryozoa Living and Fossil* (pp. 133–145). Nantes: Bulletin de la Société Sciences Naturelles de l'Ouest de la France, Mémoire HS 1.

Gordon, D. P., Ramalho, L. V., & Taylor, P. D. (2006). An unreported invasive bryozoan that can affect livelihoods - Membraniporopsis tubigera in New Zealand and Brasil. *Bulletin of Marine Science*, *78*(2), 331–342.

Gordon, D. P., & Rudman, W. B. (2006). *Integripelta acanthus* n. Sp. (Bryozoa: Eurystomellidae) - a tropical prey species of *Okenia hiroi* (Nudibranchia). *Zootaxa*, *1229*, 41–48.

Gordon, D. P., & Spencer Jones, M. E. (2013). The amathiiform Ctenostomata (phylum Bryozoa) of New Zealand - including four new species, two of them of probable alien origin. *Zootaxa*, *3647*(1), 75–95.

Gordon, D. P., & Taylor, P. D. (2017). Resolving the status of *Pyriporoides* and *Daisyella* (Bryozoa: Cheilostomata), with the systematics of some additional taxa of Callo-

poroidea having an ooecial heterozooid. *Zootaxa*, *4242*(2), 201–232. Retrieved from http://mapress.com/j/zt/article/view/zootaxa.4242.2.1

Gómez, A., Wright, P. J., Lunt, D. H., Cancino, J. M., Carvalho, G. R., & Hughes, R. N. (2007). Mating trials validate the use of DNA barcoding to reveal cryptic speciation of a marine bryozoan taxon. *Proceedings of the Royal Society of London, B*, *274*, 199–207.

Grischenko, A. V. (2002). History of investigations and current state of knowledge of bryozoan species diversity in the Bering Sea. In P. N. Wyse Jackson & M. E. Spencer Jones (Eds.), *Annals of Bryozoology: Aspects of the history of research on bryozoans* (pp. 97–116). Dublin: International Bryozoology Association.

Grischenko, A. V. (2013). First record of a bathyal bryozoan fauna from the Sea of Japan. *Deep-Sea Research II*, *86-87*, 172–180.

Grischenko, A. V. (2014). An unknown archive of German A. Kluge: A bryozoans collection in the Museum of Invertebrate Zoology at Perm State National Research University. In *Annals of Bryozoology 4: Aspects of the history of research on bryozoans.* (Vol. 4, pp. 75–92).

Grischenko, A. V., & Chernyshev, A. V. (2015). Triticella minini–a new ctenostome bryozoan from the abyssal plain adjacent to the Kuril–Kamchatka Trench. *Deep Sea Research Part II: Topical Studies in Oceanography*, *111*, 343–350.

Grischenko, A. V., Gordon, D. P., & Taylor, P. D. (1998). A unique new genus of cheilostomate bryozoan with reversed-polarity zooidal budding. *Asian Marine Biology*, *15*, 105–117.

Grischenko, A. V., & Mawatari, S. F. (2006). On the bryozoan diversity of Sagami Bay. *Mem Nat Sci Mus Tokyo*, *40*, 187–201.

Grischenko, A. V., Taylor, P. D., & Mawatari, S. F. (2004). Doryporella smirnovi sp. Nov. (Bryozoa: Cheilostomata) and its impact on phylogeny and classification. *Zoological Science*, *21*, 327–332.

Grischenko, A. V., & Zvyagintsev, A. Y. (2012). On the state of inventory of the bryozoan fauna in Peter the Great Bay of the Sea of Japan in light of detection of the cheilostome bryozoans *Callopora sarae* and *Microporella trigonellata*. *Russian Journal of Biological Invasions*, *3*(3), 172–179.

Gruhl, A. (2008). Muscular systems in gymnolaemate bryozoan larvae (Bryozoa: Gymnolaemata). *Zoomorphology*, *127*(3), 143–159. https://doi.org/10.1007/s00435-008-0059-3

Gruhl, A. (2010). Ultrastructure of mesoderm formation and development in Membranipora membranacea (Bryozoa: Gymnolaemata). *Zoomorphology*, *129*(1), 45–60.

Grunbaum, D. (1997). Hydromechanical mechanisms of colony organization and cost of defense in an encrusting bryozoan, Membranipora membranacea. *Limnology and Oceanography*, *42*(4), 741–752. Retrieved from://000071037700012

Hall, D. N. (1982). Larval release in Celleporaria apiculata (Busk) (Bryozoa: Ascophora). *Journal of Natural History*, *16*, 195–200.

Hamond, R. (1975). The marine and brackish-water Bryozoa of Norfolk. *Transactions of the Norfolk and Norwich Naturalists' Society*, *22*(6), 406–423.

Hara, U. (2000). Bryozoan fragments from Eocene glacial erratics of McMurdo Sound, East Antarctica. In J. D. Stilwel & R. M. Feldman (Eds.), *Paleobiology and paleoenvironments of eocene fossiliferous erratics, mcmurdo sound, east antarctica, geophysical union, antarctic research series* (Vol. 76, pp. 321–323).

Harmelin, J.-G. (2014). *Monoporella bouchardii* (Audouin & Savigny, 1826) (Bryozoa, Cheilostomata): A forgotten taxon redescribed from Eastern Mediterranean material. *Cahiers de Biologie Marine*, *55*, 91–99.

Harmelin, J.-G. (2017). Bryozoan facies in the coralligenous community: Two assemblages with contrasting features at Port-Cros Archipelago (Port-Cros National Park, France, Mediterranean). *Scientific Reports of the Port-Cros National Park*, *31*, 105–123.

Harmelin, J.-G., Bitar, G., & Zibrowius, H. (2009). Smittinidae (Bryozoa, Cheilostomata) from coastal habitats of Lebanon (Mediterranean Sea), including new and non-indigenous species. *Zoosystema*, *31*(1), 163–187.

Harmelin, J.-G., Bitar, G., & Zibrowius, H. (2016). High xenodiversity versus low native diversity in the south-eastern Mediterranean: Bryozoans from the coastal zone of Lebanon. *Mediterranean Marine Science*, *17*(2), 417–439.

Harmelin, J.-G., & d'Hondt, J.-L. (1993). Transfers of bryozoan species between the Atlantic Ocean and the Mediterranean Sea via the Strait of Gibraltar. *Oceanologica Acta*, *16*(1), 63–72.

Harmelin, J.-G., Ostrovsky, A. N., Caceres-Chamizo, J. P., & Sanner, J. (2011). Bryodiversity in the tropics: Taxonomy of *Microporella* species (Bryozoa, Cheilostomata) with personate maternal zooids from Indian Ocean, Red Sea and southeast Mediterranean. *Zootaxa*, *2798*, 1–30.

Harmelin, J.-G., Vieira, L. M., Ostrovsky, A. N., Cáceres-Chamizo, J. P., & Sanner, J. (2012). *Scorpiodinipora costulata* (Canu & Bassler, 1929) (Bryozoa, Cheilostomata), a taxonomic and biogeographic dilemma: Complex of cryptic species or human-mediated cosmopolitan colonizer? *Zoosystema*, *34*(1), 123–138.

Hastings, A. B. (1927). Zoological results of the Cambridge Expedition to the Suez Canal 1924. Part 20. Report on the Polyzoa. *Transactions of the Zoological Society of London*, *22*, 331–354.

Hastings, A. B. (1941). The British species of Scruparia (Polyzoa). *Annals and Magazine of Natural History*, *(11)7*, 465–472.

Hastings, A. B. (1943). Polyzoa (Bryozoa) I. Scrupocellariidae, Epistomiidae, Farciminariidae, Bicellariellidae, Aeteidae, Scrupariidae. *Discovery Reports*, *22*, 301–510.

Hayward, P. J. (1976). The marine fauna and flora of the Isles of Scilly: Bryozoa II. *Journal of Natural History*, *10*, 319–330.

Hayward, P. J. (1978). Bryozoa from the west European continental slope. *Journal of Zoology, London*, *184*, 207–224.

Hayward, P. J. (1979). Deep water Bryozoa from the coasts of Spain and Portugal. *Cahiers de Biologie Marine*, *20*, 59–75.

Hayward, P. J. (1982). A new species of bryozoan epizooite from South Africa. *Journal of Natural History*, *16*, 769–773.

Hayward, P. J. (1988a). Mauritian cheilostome Bryozoa. *Journal of Zoology, London*, *215*, 269–356.

Hayward, P. J. (1988b). The Recent species of Adeonella (Bryozoa: Cheilostomata) including descriptions of fifteen new species. *Zoological Journal of the Linnean Society*, *94*, 111–191.

Hayward, P. J. (1992). Some Antarctic and sub-Antarctic species of Celleporidae (Bryozoa, Cheilostomata). *Journal of Zoology*, *226*(2), 283–310.

Hayward, P. J., & Parker, S. A. (1994). Notes on some species of Parasmittina Osburn, 1952 (Bryozoa: Cheilostomatida. *Zoological Journal of the Linnean Society*, *110*, 53–75.

Hayward, P. J., & Ryland, J. S. (1978). Bryozoa from the Bay of Biscay and Western Approaches. *Journal of the Marine Biological Association of the United Kingdom*, *58*, 143–159.

Hayward, P. J., & Ryland, J. S. (1993). Taxonomy of six Antarctic anascan Bryozoa. *Antarctic Science*, *5*(2), 129–136.

Hayward, P. J., & Ryland, J. S. (1995a). Bryozoa from Heron Island, Great Barrier Reef. 2. *Memoirs of the Queensland Museum*, *38*(2), 533–573.

Hayward, P. J., & Ryland, J. S. (1995b). The British species of Schizoporella (Bryozoa: Cheilostomatida). *Journal of Zoology, London*, *237*, 37–47.

Hayward, P. J., & Ryland, J. S. (1998). *Cheilostomatous Bryozoa. Part 1. Aeteoidea - Cribrilinoidea.* Shrewsbury: Field Studies Council.

Hayward, P. J., & Thorpe, J. P. (1988). Species of Chaperiopsis (Bryozoa, Cheilostomata) collected by Discovery Investigations. *Journal of Natural History*, *22*, 45–69.

Hayward, P. J., & Thorpe, J. P. (1989). Membraniporoidea, Microporoidea and Cellarioidea (Bryozoa, Cheilostomata) collected by Discovery Investigations. *Journal of Natural History*, *23*, 913–959.

Hayward, P. J., & Thorpe, J. P. (1995). Some British species of Schizomavella (Bryozoa: Cheilostomatida). *Journal of Zoology, London*, *235*, 661–676.

Hayward, P. J., & Winston, J. E. (2011). Bryozoa collected by the United States Antarctic Research Program: New taxa and new records. *Journal of Natural History*, *45*(37-38), 2259–2338.

Heindl, H., Thiel, V., Wiese, J., & Imhoff, J. F. (2012). Bacterial isolates from the bryozoan *Membranipora membranacea*: Influence of culture media on isolation and antimicrobial activity. *International Microbiology*, *15*, 17–32. https://doi.org/10.2436/20.1501.01.155

Helmkampf, M., Bruchhaus, I., & Hausdorf, B. (2008). Multigene analysis of lophophorate and chaetognath phylogenetic relationships. *Molecular Phylogenetics and Evolution*, *46*(1), 206–214.

Herrera-Cubilla, A., Dick, M. H., Sanner, J., & Jackson, J. B. C. (2006). Neogene Cupuladriidae of tropical America. I: Taxonomy of Recent *Cupuladria* from opposite sides of the Isthmus of Panama. *Journal of Paleontology*, *80*(2), 245–263.

Herrera-Cubilla, A., Dick, M. H., Sanner, J., & Jackson, J. B. C. (2008). Neogene Cupuladriidae of tropical America. II: Taxonomy of Recent *Discoporella* from opposite sides of the Isthmus of Panama. *Journal of Paleontology*, *82*(2), 279–298.

Hincks, T. (1860). Descriptions of new Polyzoa from Ireland. *Quarterly Journal of Microscopical Science*, *8*(32), 275–280.

Hincks, T. (1882). Report on the Polyzoa of the Queen Charlotte Islands. *Annals and Magazine of Natural History*, *10*, 459–471.

Hincks, T. (1886). The Polyzoa of the Adriatic, I. *Annals and Magazine of Natural History*, *(5)17*, 254–271.

Hirose, M. (2011). Orientation and righting behavior of the sand-dwelling bryozoan *Conescharellina catella*. *Invertebrate Biology*, *130*(3), 282–290. https://doi.org/10.1111/j.1744-7410.2011.00237.x

Hirose, M. (2012). Revision of the genus *Buchneria* (Bryozoa, Cheilostomata) from Japan. *Zookeys*, *241*, 1–19.

Hirose, M. (2017). Diversity of freshwater and marine bryozoans in Japan. In M. Motokawa & H. Kajihara (Eds.), *Species Diversity of Animals in Japan* (pp. 629–649). Springer.

Hirose, M., Mawatari, S. F., & Scholz, J. (2012). Distribution and diversity of erect bryozoan assemblages along the Pacific coast of Japan. In A. Ernst, P. Schäfer, & J. Scholz (Eds.), *Bryozoan Studies 2010* (Vol. 143, pp. 121–136). Berlin: Springer.

Hu, Y.-m., & Wang, F.-z. (1984). A new species of Ectoprocta from the Antarctic. *Acta Zootaxonomica Sinica*, *9*, 241–243.

Huang, Z., Li, C., & Liu, X. (1990). The bryozoan foulers of Hong Kong and neighbouring waters. In B. Morton (Ed.), *The marine flora and fauna of Hong Kong and southern China, 1986.: Vol. 2. Taxonomy and ecology* (pp. 737–765). Hong Kong: Hong Kong University Press.

Hughes, D. J., & Jackson, J. B. C. (1992). Distribution and abundance of cheilostome bryozoans on the Caribbean reefs of central Panama. *Bulletin of Marine Science*, *51*(3), 443–465.

Hughes, R. L., & Woollacott, R. M. (1978). Ultrastructure of potential photoreceptor organs in the larva of *Scrupocellaria bertholetti* (Bryozoa). *Zoomorphologie*, *91*, 225–234.

Hughes, R. L., & Woollacott, R. M. (1980). Photoreceptors of bryozoan larvae (Cheilostomata, Cellularioidea). *Zoologica Scripta*, *9*, 129–138.

Hughes, R. N., & Wright, P. J. (2014). Self-fertilisation in the Celleporella angusta clade and a description of Celleporella osiani sp. Nov. *Studi Trentini Di Scienze Naturali*, *94*, 119–124.

Hunter, E., Okano, K., Tomono, Y., & Fusetani, N. (1998). Functional partitioning of energy reserves by larvae of the marine bryozoan *Bugula neritina* (L.). *Journal of Experimental Biology*, *201*, 2857–2865. Retrieved from://000077039700007

Igić, L. (n.d.). *Some species of bryozoa from the Adriatic sea and from freshwaters, which are of special importance for fouling complex.* Center for Investigation of Sea "Ruđer Bošković", Rovinj, Croatia.

Ito, M., Onishi, T., & Dick, M. H. (2015). Cribrilina mutabilis n. Sp., an eelgrass-associated bryozoan (Gymnolaemata: Cheilostomata) with large variation in zooid morphology related to life history. *Zoological Science*, *32*, 485–497.

Iyengar, E. V., & Harvell, C. D. (2002). Specificity of cues inducing defensive spines in the bryozoan Membranipora membranacea. *Marine Ecology-Progress Series*, *225*, 205–218.

Javed, M., & Tirmizi, N. M. (1993). Four species of Bugula (Bryozoa: Bugulidae) new to the Pakistan coast (northern Arabian Sea). *Pakistan Journal of Zoology*, *25*(4), 285–288.

Jelly, E. C. (1889). *A synonymic catalogue of the Recent marine Bryozoa including fossil synonyms.* London: Dulau & Company.

Johnson, C. H. (2010). Effects of Selfing on Offspring Survival and Reproduction in a Colonial Simultaneous Hermaphrodite (Bugula Stolonifera, Bryozoa). *The Biological Bulletin*, *219*(1), 27–37.

Johnson, C. H., Winston, J. E., & Woollacott, R. M. (2012). Western Atlantic introduction and persistence of the marine bryozoan Tricellaria inopinata. *Aquatic Invasions*, *7*(3), 295–303.

Jr, G. R. M., & McKinney, F. K. (2000). A Theoretical Morphologic Analysis of Convergently Evolved Erect Helical Colony Form in the Bryozoa. *Paleobiology*, *26*(4), 556–577. https://doi.org/10.1666/0094-8373(2000)026%3C0556:atmaoc%3E2.0.co;2

Kaiser, S., Barnes, D. K. A., Linse, K., & Brandt, A. (2008). Epibenthic macrofauna associated with the shelf and slope of a young and isolated Southern Ocean island. *Antarctic Science*, *20*(3), 281–290.

Karagodina, N. P., Vishnyakov, A. E., Kotenko, O. N., Maltseva, A. L., & Ostrovsky, A. N. (2018). Ultrastructural evidence for nutritional relationships between a marine colonial invertebrate (Bryozoa) and its bacterial symbionts. *Symbiosis*, 1–10.

Kasaei, S. M., Nasrolahi, A., Abtahi, B., & Taylor, P. D. (2017). Bryozoa of the southern Caspian Sea, Iranian coast. *Check List*, *13*(4), 305–313. https://doi.org/10.15560/13.4.305

Kaselowsky, J., Scholz, J., & Levit, G. S. (2002). The biological potential of encrusting bryozoans. *Senckenbergiana Lethaea*, *82*(1), 181–192.

Kaselowsky, J., Scholz, J., Mawatari, S. F., Probert, P. K., Gerdes, G., Kadagies, N., & Hillmer, G. (2005). Bryozoans and microbial communities of cool-temperate to subtropical latitudes - paleoecological implications. I. Growth morphologies of shallow-water bryozoans settling on bivalve shells (Japan and New Zealand). *Facies*, *50*, 349–361.

Kelmo, F., Attrill, M. J., Gomes, R. C. T., & Jones, M. B. (2004). El Niño induced local extinction of coral reef bryozoan species from Northern Bahia, Brazil. *Biological Conservation*, *118*, 609–617.

Kelso, A., & Wyse Jackson, P. N. (2012). Invasive bryozoans in Ireland: First record of Watersipora subtorquata (d'Orbigny, 1852) and an extension of the range of *Tricellaria inopinata* d'Hondt and *Occhipinti ambrogi*, 1985. *BioInvasions Records*, *1*(3), 209–214.

Kelso, A., & Wyse Jackson, P. N. (2014). Albert Russell Nichols (1859-1933) and bryozoan research in Ireland during the late 19th and early 20th centuries. *International Bryozoology Association, Dublin*, 93–106.

Key, M. M., Hollenbeck, P. M., O'Dea, A., & Patterson, W. P. (2013). Stable isotope profiling in modern marine bryozoan colonies across the Isthmus of Panama. *Bulletin of Marine Science*, *89*(4), 837–856.

Kind, B., De Blauwe, H., Faasse, M., & Kuhlenkamp, R. (2015). *Schizobrachiella verrilli* (Bryozoa, Cheilostomata) new to Europe. *Marine Biodiversity Records*, *8*.

Kind, B., & Kuhlenkamp, R. (2018). Discovery of the non-indigenous bryozoan Smittoidea prolifica Osburn, 1952 near Helgoland: First record in 2011 for the German North Sea. *Marine Biodiversity*, *48*(2), 1237–1240.

Kobluk, D. R., Cuffey, R. J., Fonda, S. S., & Lysenko, M. A. (1988). Cryptic Bryozoa, leeward fringing reef of Bonaire, Netherlands Antilles, and their paleoecological application. *Journal of Paleontology*, *62*(3), 427–439.

Koçak, F. (2007). A new alien bryozoan *Celleporaria brunnea* (Hincks, 1884) in the Aegean Sea (eastern Mediterranean). *Scientia Marina*, *71*(1), 191–195.

Koçak, F., Baldu, A., i, & Benli, H. A. (2002). Epiphytic bryozoan community of *Posidonia oceanica* (L.) Delile meadow in the northern Cyprus (Eastern Mediterranean). *Indian Journal of Marine Sciences*, *31*(3), 235–238.

Koçak, F., & Önen, A. (2014). Checklist of Bryozoa on the coasts of Turkey. *Turkish Journal of Zoology*, *38*, 880–891.

Kozloff, E. N., & Price, L. H. (1996). *Marine Invertebrates of the Pacific Northwest* (Rep Sub). Seattle: Univ of Washington Press.

Krzeminska, M., Sicinski, J., & Kuklinski, P. (2018). Biodiversity and biogeographic affiliation of Bryozoa from King George Island (Antarctica). *Systematics and Biodiversity*, *16*(6), 576–586.

Kubota, K., & Mawatari, S. F. (1985). A systematic study of cheilostomatous bryozoans from Oshoro Bay, Hokkaido : 1. Anasca. *Environmental Science, Hokkaido*, *8*(1), 75–91.

Kubota, K., & Mawatari, S. F. (1986). A systematic study of ceilostomatous bryozoans from Oshoro Bay, Hokkaido. 2. Ascophora. *Environmental Science, Hokkaido*, *8*(2), 195–208.

Kuklinski, P. (2002). Fauna of Bryozoa from Kongsfjorden, West Spitsbergen. *Polish Polar Research*, *23*(2), 193–206.

Kuklinski, P. (2009). Ecology of stone-encrusting organisms in the Greenland Sea - a review. *Polar Research*, *28*, 222–237.

Kuklinski, P., & Bader, B. (2007). Comparison of bryozoan assemblages from two contrasting Arctic shelf regions. *Estuarine, Coastal and Shelf Science*, *73*(3-4), 835–843. https://doi.org/10.1016/j.ecss.2007.03.024

Kuklinski, P., & Barnes, D. K. A. (2009). A new genus and three new species of Antarctic cheilostome Bryozoa. *Polar Biology*, *32*, 1251–1259.

Kuklinski, P., Grischenko, A. V., & Jewett, S. C. (2015). Two new species of the cheilostome bryozoan Cheilopora from the Aleutian Islands. *Zootaxa*, *3963*(3), 434–442.

Kuklinski, P., & Hayward, P. J. (2004). Two new species of cheilostome Bryozoa from Svalbard. *Sarsia*, *89*, 79–84.

Kuklinski, P., & Taylor, P. D. (2006). A new genus and some cryptic species of Arctic and boreal calloporid cheilostome bryozoans. *Journal of the Marine Biological Association*, *86*, 1035–1046.

Kuklinski, P., & Taylor, P. D. (2007). Are bryozoans adapted for living in the Arctic? *Proceedings of the 14th International Bryozoology Association Conference, Boone, North Carolina, July 1-8, 2007, Virginia Museum of Natural History. Special Publication No. 15*, 101–110.

Kuklinski, P., & Taylor, P. D. (2008). Arctic species of the cheilostome bryozoan Microporella, with a redescription of the type species. *Journal of Natural History*, *42*(27-28), 1893–1906.

Kuklinski, P., Taylor, P. D., & Denisenko, N. V. (2007). Arctic cheilostome bryozoan species of the genus *Escharoides*. *Journal of Natural History*, *41*(1-4), 219–228.

Kutyumov, V. A., Maltseva, A. L., Kotenko, O. N., & Ostrovsky, A. N. (2016). Functional differentiation in bryozoan colonies: A proteomic analysis. *Cell and Tissue Biology*, *10*(2), 152–159. https://doi.org/10.1134/S1990519X16020073

Lacourt, A. W. (1949). Bryozoa of the Netherlands. *Archives Nederlandaises de Zoologie*, *8*, 289–321.

Landsborough, D. (1852). *A Popular History of British Zoophytes, or Corallines.* London: Reeve and Co.

Lauer, A. (2016). *Watersipora subtorquata* and the possible role of its associated microbes: An attempt to explain the extraordinary invasion success of this marine bryozoan species. In C. J. Hurst (Ed.), *The Mechanistic Benefits of Microbial Symbionts* (Vol.

2, pp. 239–268). Cham: Springer International Publishing. https://doi.org/10.1007/978-3-319-28068-4_9

Lepoint, G., Mouchette, O., Pelaprat, C., & Gobert, S. (2014). An ecological study of *Electra posidoniae* Gautier, 1954 (Cheilostomata, Anasca), a bryozoan epiphyte found solely on the seagrass *Posidonia oceanica* (L.) Delile, 1813. *Belgian Journal of Zoology, 144*(1), 51–63.

Lim, G. E., & Haygood, M. G. (2004). "Candidatus *Endobugula glebosa*," a specific bacterial symbiont of the marine bryozoan *Bugula* simplex. *Applied and Environmental Microbiology, 70*(8), 4921–4929.

Linneman, J., Paulus, D., Lim-Fong, G. E., & Lopanik, N. B. (2014). Latitudinal variation of a defensive symbiosis in the *Bugula neritina* (Bryozoa) sibling species complex. *PLoS One, 9*(10). https://doi.org/10.1371/journal.pone.0108783

Linse, K., Barnes, D. K. A., & Enderlein, P. (2006). Body size and growth of benthic invertebrates along an Antarctic latitudinal gradient. *Deep-Sea Research II, 53*, 921–931.

Liu, X.-X. (1992). On the genus Membranipora (Anasca: Cheilostomata: Bryozoa) from south Chinese seas. *Raffles Bulletin of Zoology, 40*(1), 103–144.

Liuzzi, M. G., López-Gappa, J., & Salgado, L. (2018). Bryozoa from the continental shelf off Tierra del Fuego (Argentina): Species richness, colonial growth-forms, and their relationship with water depth. *Estuarine, Coastal and Shelf Science, 214*, 48–56. https://doi.org/10.1016/j.ecss.2018.09.014

Livingstone, A. A. (1924). Studies on Australian Bryozoa. No.1. *Records of the Australian Museum, 14*, 189–212.

Livingstone, A. A. (1928). The Bryozoa. Supplementary Report. *Scientific Reports of the Australian Antarctic Expedition 1911-1914, Series C, Zoology and Botany, 9*(1), 1–93.

Lodola, A., Savini, D., & Occhipinti-Ambrogi, A. (2012). First record of *Tricellaria inopinata* (Bryozoa: Candidae) in the harbours of La Spezia and Olbia, Western Mediterranean Sea (Italy). *Marine Biodiversity Records, 5.*

Lomas, J. (1886). Report on the Polyzoa of the L. M. B. C. District. In *Report upon the fauna of Liverpool Bay and the neighboring seas* (Vol. 1, pp. 161–200). London: Liverpool Marine Biology Committee, University College Liverpool.

Lombardi, C., Cocito, S., Gambi, M. C., Cisterna, B., Flach, F., Taylor, P. D., … Cusack, M. (2011). Effects of ocean acidification on growth, organic tissue and protein profile of the Mediterranean bryozoan Myriapora truncata. *Aquatic Biology, 13*(3), 251–262.

Lombardi, C., Gambi, M. C., Vasapollo, C., Taylor, P. D., & Cocito, S. (2011). Skeletal alterations and polymorphism in a Mediterranean bryozoan at natural CO2 vents. *Zoomorphology, 130*, 135–145.

Lombardi, C., Rodolfo-Metalpa, R., Cocito, S., Gambi, M. C., & Taylor, P. D. (2011). Structural and geochemical alterations in the Mg calcite bryozoan LiverpoolMyriapora truncata under elevated seawater pCO2 simulating ocean acidification. *Marine Ecology, 32*(2), 211–221.

Lombardi, C., Taylor, P. D., & Cocito, S. (2014). Bryozoan constructions in a changing mediterranean sea. In *The Mediterranean Sea* (pp. 373–384). Springer, Dordrecht. https://doi.org/10.1007/978-94-007-6704-1_21

Lombardi, C., Taylor, P. D., Cocito, S., Bertolini, C., & Calosi, P. (2017). Low pH conditions impair module capacity to regenerate in a calcified colonial invertebrate, the bryozoan *Cryptosula pallasiana*. *Marine Environmental Research*, *125*, 110–117. https://doi.org/10.1016/j.marenvres.2017.02.002

Long, E. R., & Rucker, J. B. (1960). A comparative study of cheilostome Bryozoa at Yokosuka, Maizuru, and Sasebo, Japan. *Pacific Science*, *23*, 56–69.

Long, E. R., & Rucker, J. B. (1970). Offshore marine cheilostome Bryozoa from Fort Lauderdale, Florida. *Marine Biology*, *6*, 18–25.

Lopanik, N., Lindquist, N., & Targett, N. (2004). Potent cytotoxins produced by a microbial symbiont protect host larvae from predation. *Oecologia*, *139*, 131–139.

Loxton, J., Kuklinski, P., Mair, J. M., Spencer Jones, M. E., & Porter, J. S. (2012). Patterns of magnesium-calcite distribution in the skeleton of some polar bryozoan species. In A. Ernst, P. Schäfer, & J. Scholz (Eds.), *Bryozoan Studies 2010* (Vol. 143, pp. 169–185). Berlin: Springer.

Loxton, J., Wood, C. A., Bishop, J. D. D., Porter, J. S., Jones, M. S., & Nall, C. R. (2017). Distribution of the invasive bryozoan *Schizoporella japonica* in Great Britain and Ireland and a review of its European distribution. *Biological Invasions*, *19*(8), 2225–2235.

López de la Cuadra, C. M., & García-Gómez, J. C. (1991). A new species of *Hemicyclopora* (Bryozoa, Cheilostomata) from the southern coast of Spain. In F. P. Bigey (Ed.), *Bryozoaires Actuels et Fossiles: Bryozoa Living and Fossil* (pp. 213–218). Nantes: Bulletin de la Société Sciences Naturelles de l'Ouest de la France, Mémoire HS 1.

López de la Cuadra, C. M., & García-Gómez, J. C. (1993). Little-known Atlantic cheilostome bryozoans at the entrance to the Mediterranean. *Journal of Natural History*, *27*(2), 457–469.

López de la Cuadra, C. M., & García-Gómez, J. C. (1994). Bryozoa Cheilostomata: The genus *Amphiblestrum* in the Western Mediterranean and the first of Atlantic waters. *Journal of Natural History*, *28*, 683–693.

López de la Cuadra, C. M., & García-Gómez, J. C. (1997). Studies on Recent Macroporidae (Bryozoa: Cheilostomatida), with new taxa and ontogeny of the ovicells. *Journal of Zoology, London*, *242*, 605–621.

López de la Cuadra, C. M., & García-Gómez, J. C. (2000). The cheilostomate Bryozoa (Bryozoa: Cheilostomatida) collected by the Spanish 'Antártida 8611' Expedition to the Scotia Arc and South Shetland Islands. *Journal of Natural History*, *34*, 755–772.

López de la Cuadra, C. M., & García-Gómez, J. C. (2001). New and little known ascophoran bryozoans from the Western Mediterranean, collected by 'Fauna Ibérica' expeditions. *Journal of Natural History*, *35*, 1717–1732.

López-Fé, C. M. (2006). Some bathyal cheilostome Bryozoa (Bryozoa, Cheilostomata) from the Canary Islands (Spain, Eastern Atlantic), with descriptions of three new species, a new genus, and a new family. *Journal of Natural History*, *40*(29-31), 1801–1812. Retrieved from http://dx.doi.org/10.1080/00222930601043763

López Gappa, J. J. (1986). A new bryozoan genus from the Weddell Sea, Antarctica. *Polar Biology*, *6*, 103–105.

López Gappa, J. J. (1989). Overgrowth competition in an assemblage of encrusting bryozoans settled on artificial substrata. *Marine Ecology Progress Series*, *51*, 121–130.

López Gappa, J. J., & Landoni, N. A. (2009). Space utilisation patterns of bryozoans on the Patagonian scallop Psychrochlamys patagonica. *Scientia Marina*, *73*(1), 161–171.

López Gappa, J. J., & Liuzzi, M. G., i. (2009). A new Antarctic *Osthimosia* (Bryozoa, Cheilostomata, Celleporidae) with dimorphic zooids. *Polar Biology*, *32*, 47–51.

López-Gappa, J., & Liuzzi, M. G. (2018). Recent discovery of non-indigenous bryozoans in the fouling assemblage of Quequén Harbour (Argentina, Southwest Atlantic). *Marine Biodiversity*, *48*(2), 1159–1167.

López-Gappa, J., Pérez, L. M., & Griffin, M. (2017). First record of a fossil selenariid bryozoan in South America. *Alcheringa*, *41*(3), 365–368.

Lörz, A.-N., Myers, A., & Gordon, D. P. (2014). An inquiline deep-water bryozoan/amphipod association from New Zealand, including the description of a new genus and species of Chevaliidae. *European Journal of Taxonomy*, *72*, 1–17.

Lyke, E. B., Reed, C. G., & Woollacott, R. M. (1983). Origin of the cystid epidermis during the metamorphosis of three species of gymnolaemate bryozoans. *Zoomorphology*, *102*(2), 99–110.

MacGillivray, P. H. (1887a). A catalogue of the marine Polyzoa of Victoria. *Transactions and Proceedings of the Royal Society of Victoria*, *23*, 187–224.

MacGillivray, P. H. (1887b). Descriptions of new or little-known Polyzoa, Part 12. *Transactions and Proceedings of the Royal Society of Victoria*, *23*, 179–186.

Mackie, J. A., Darling, J. A., & Geller, J. B. (2012). Ecology of cryptic invasions: Latitudinal segregation among Watersipora (Bryozoa) species. *Scientific Reports*, *2*(871), 1–10. https://doi.org/DOI%2010.1038/srep00871

Madurell, T., Zabala, M., Dominguez-Carrió, C., & Gili, J. M. (2013). Bryozoan faunal composition and community structure from the continental shelf off Cap de Creus (Northwestern Mediterranean). *Journal of Sea Research*, *83*, 123–136.

Maltseva, A. L., Kotenko, O. N., Shabalin, K. A., Shavarda, A. L., Winson, M. K., & Ostrovsky, A. N. (2014). Novel brominated fungicidal alkaloid isolated from the marine bryozoan *Chartella membranaceatruncata* (Smitt, 1868). *Studi Trent. Sci. Nat*, *94*, 163–168.

Maplestone, C. M. (1900). Further descriptions of the Tertiary Polyzoa of Victoria. 4. *Proceedings of the Royal Society of Victoria (New Series)*, *13*, 1–9.

Maplestone, C. M. (1904). Tabulated list of the fossil cheilostomatous Polyzoa in the Victorian Tertiary deposits. *Proceedings of the Royal Society of Victoria (New Series)*, *17*, 182–219.

Maplestone, C. M. (1909). Polyzoa from the Gilbert Islands. *Proceedings of the Royal Society of Victoria (New Series)*, *21*, 410–419.

Maplestone, C. M. (1910). On a new species of Cellepora from the south Australian coast. *Proceedings of the Royal Society of Victoria (New Series)*, *23*, 39–41.

Marchini, A., Cunha, M. R., & Occhipinti Ambrogi, A. (2007). First observations on bryozoans and entoprocts in the Ria de Aveiro (NW Portugal) including the first record of the Pacific invasive cheilostome Tricellaria inopinata. *Marine Ecology*, *28*(Suppl. 1), 154–160.

Markert, A., Matsuyama, K., Rohde, S., Schupp, P., & Wehrmann, A. (2016). First record of the non-native Pacific bryozoan *Smittoidea prolifica* Osburn, 1952 at the German North Sea coast. *Marine Biodiversity*, *46*(3), 717–723.

Marques, A. C., Kloh, A. dos S., Migotto, A. E., Cabral, A. C., Rigo, A. P. R., Bettim, A. L., … Kremer, L. P. (2013). Rapid assessment survey for exotic benthic species in the São Sebastião Channel, Brazil. *Latin American Journal of Aquatic Research*, *41*(2), 398–407.

Marshall, D. J., Bolton, T. F., & Keough, M. J. (2003). Offspring size affects the post-metamorphic performance of a colonial marine invertebrate. *Ecology*, *84*(12), 3131–3137.

Mathew, M., & Lopanik, N. B. (2014). Host Differentially Expressed Genes During Association With Its Defensive Endosymbiont. *The Biological Bulletin*, *226*(2), 152–163.

Matsuyama, K., Janssen, A., Martínez Arbizu, P., Martha, S. O., & Freiwald, A. (2014). Bryozoans from RV Sonne deep-sea cruises SO 167 'Louisville' and SO 205 'Mangan'. *Zootaxa*, *3856*(1), 100–116.

Matsuyama, K., Titschack, J., Baum, D., & Freiwald, A. (2015). Two new species of erect Bryozoa (Gymnolaemata: Cheilostomata) and the application of non-destructive imaging methods for quantitative taxonomy. *Zootaxa*, *4020*(1), 81–100.

Maturo, F. J. S., & Schopf, T. J. M. (1968). Ectoproct and entoproct type material: Reexamination of species from New England and Bermuda named by A.E. Verrill, J.W. Dawson and E. Desor. *Postilla*, *120*, 1–95.

Mawatari, S. F., & Suwa, T. (1998). Two new species of Japanese *Microporella* (Bryozoa, Cheilostomatida) in the Döderlein Collection, Musée Zoologique, Strasbourg. *Cahiers de Biologie Marine*, *39*(1), 1–7.

McCann, L. (2019). Bryozoa (Cheilostomata, Ctenostomata, and Cyclostomata) in Galapagos Island Fouling Communities. *Aquatic Invasions*, *14*(1), 85–131. https://doi.org/10.3391/ai.2019.14.1.04

McCann, L. D., Gray Hitchcock, N., Winston, J. E., & Ruiz, G. M. (2007). Non-native bryozoans in coastal embayments of the southern United States: New records for the Western Atlantic. *Bulletin of Marine Science*, *80*(2), 319–342.

McCuller, M. I., & Carlton, J. T. (2018). Transoceanic rafting of Bryozoa (Cyclostomata, Cheilostomata, and Ctenostomata) across the North Pacific Ocean on Japanese tsunami marine debris. *Aquatic Invasions*, *13*(1), 137–162.

McCuller, M. I., Carlton, J. T., & Geller, J. B. (2018). *Bugula tsunamiensis* n. Sp. (Bryozoa, Cheilostomata, Bugulidae) from Japanese tsunami marine debris landed in the Hawaiian Archipelago and the Pacific Coast of the USA. *Aquatic Invasions*, *13*(1), 163–172.

McGhee, G., Jr. (2000). A theoretical morphologic analysis of convergently evolved erect helical colony form in the Bryozoa. *Paleobiology*, *26*(4), 556–577. https://doi.org/10.1666/0094-8373(2000)026%3C0556:ATMAOC%3E2.0.CO;2

McKinney, F. K., & Jaklin, A. (2000). Spatial niche partitioning in the *Cellaria* meadow epibiont association, northern Adriatic Sea. *Cahiers de Biologie Marine*, *41*(1), 1–17.

McKinney, F. K., & McKinney, M. J. (1993). Larval behaviour and choice of settlement site: Correlation with environmental distribution pattern in an erect bryozoan. *Facies*, *29*, 119–132.

Menon, N. R. (1972). Species of the genus Scrupocellaria Van Beneden (Bryozoa, Anasca) from Indian waters. *Internationale Revue Der Gesamten Hydrobiologie Und Hydrographie*, *57*(6), 913–931.

Menon, N. R., & Nair, N. B. (1982). Indian species of Malacostega (Polyzoa, Ectoprocta). *The Marine Biological Association of India*, *17*, 553–579.

Metcalfe, K., Gordon, D. P., & Hayward, E. (2007). An amphibious bryozoan from living mangrove leaves - *Amphibiobeania* new genus (Beaniidae). *Zoological Science*, *24*, 563–570.

Micael, J., Jardim, N., Núñez, C., Occhipinti Ambrogi, A., & Costa, A. C. (2016). Some Bryozoa species recently introduced into the Azores: Reproductive strategies as a proxy for further spread. *Helgoland Marine Research*, *70*(7). https://doi.org/10.1186/s10152-016-0458-7

Micael, J., Marina, J. G., Costa, A. C., & Occhipinti-Ambrogi, A. (2014). The non-indigenous *Schizoporella errata* (Bryozoa: Cheilostomatida) introduced into the Azores Archipelago. *Marine Biodiversity Records*, *7*, e133.

Micael, J., Tempera, F., Berning, B., López-Fé, C. M., Occhipinti-Ambrogi, A., & Costa, A. C. (2017). Shallow-water bryozoans from the Azores (central North Atlantic): Native vs. Non-indigenous species, and a method to evaluate taxonomic uncertainty. *Marine Biodiversity*, 1–12. https://doi.org/10.1007/s12526-017-0833-x

Micael, J., Tempera, F., Berning, B., López-Fé, C. M., Occhipinti-Ambrogi, A., & Costa, A. C. (2019). Shallow-water bryozoans from the Azores (central North Atlantic): Native vs. Non-indigenous species, and a method to evaluate taxonomic uncertainty. *Marine Biodiversity*, *49*(1), 469–480. https://doi.org/10.1007/s12526-017-0833-x

Min, B. S., Seo, J. E., Grischenko, A. V., & Gordon, D. P. (2017). Intertidal Bryozoa from Korea—new additions to the fauna and a new genus of Bitectiporidae (Cheilostomata) from Baengnyeong Island, Yellow Sea. *Zootaxa*, *4226*(4), 451–470.

Min, B. S., Seo, J. E., Grischenko, A. V., Lee, S.-K., & Gordon, D. P. (2017). Systematics of some calloporid and lacernid Cheilostomata (Bryozoa) from coastal South Korean waters, with the description of new taxa. *Zootaxa*, *4226*(4), 471–486.

Miranda, A. A., Almeida, A. C. S., & Vieira, L. M. (2018). Non-native marine bryozoans (Bryozoa: Gymnolaemata) in Brazilian waters: Assessment, dispersal and impacts. *Marine Pollution Bulletin*, *130*, 184–191. https://doi.org/10.1016/j.marpolbul.2018.03.023

Moosbrugger, M., Schwaha, T., Walzl, M. G., Obst, M., & Ostrovsky, A. N. (2012). The placental analogue and the pattern of sexual reproduction in the cheilostome bryozoan *Bicellariella ciliata* (Gymnolaemata). *Frontiers in Zoology*, *9*(1), 1–20.

Morgado, E. H., & Tanaka, M. O. (2001). The macrofauna associated with the bryozoan *Schizoporella errata* (Waters) in southeastern Brazil. *Scientia Marina*, *65*(3), 173–181.

Morris, B. F., & Mogelberg, D. D. (1973). Identification manual to the pelagic Sargassum fauna. *Special Publication of the Bermuda Biological Station for Research*, *11*, 1–63.

Morris, R. H., Abbott, D. P., & Haderlie, E. C. (1980). Bryozoa and Entoprocta: The Moss Animals. In *Intertidal invertebrates of California* (pp. 91–107). Stanford, Calif.: Stanford University Press.

Morrison, M., Jones, E. G., Consalvey, M., & Berkenbusch, K. (2014). *Linking marine fisheries species to biogenic habitats in New Zealand: A review and synthesis of knowledge*. New Zealand: Ministry for Primary Industries.

Moyano, H. I. (1982). Magellanic Bryozoa: Some ecological and zoogeographical aspects. *Marine Biology*, *67*, 81–96.

Moyano, H. I. (1999). Magellan Bryozoa: A review of the diversity and of the Subantarctic and Antarctic zoogeographical links. *Scientia Marina*, *63*, 219–226.

Moyano, H. I. (2011). On *Xenoflustra voigti* n. Gen., n. Sp. (Bryozoa, Cheilostomatida, Buguloidea), a new flustrine bryozoan from the south western Atlantic Ocean. *Anales Instituto Patagonia*, *39*(2), 67–71.

Moyano, H. I., & Gordon, D. P. (1980). New species of Hippothoidae (Bryozoa) from Chile, Antarctica and New Zealand. *Journal of the Royal Society of New Zealand*, *10*(1), 75–95.

Moyano G., H. I. (1986). Bryozoa marinos chilenos VI. Cheilostomata Hippothoidae: South eastern Pacific species. *Boletín de La Real Sociedad Española de Historia Natural*, *57*, 89–135.

Moyano G., H. I. (2002). Towards a general history of the south eastern Pacific bryozoans. In P. N. Wyse Jackson & M. E. Spencer Jones (Eds.), *Annals of Bryozoology: Aspects of the history of research on bryozoans* (pp. 171–183). Dublin: International Bryozoology Association.

Muravchik, M., & Griffin, M. (2004). Bryozoans from the Paraná Formation (Miocene), in Entre Ríos province, Argentina. *Ameghiniana*, *41*(1), 3–12.

Nall, C. R., Guerin, A. J., & Cook, E. J. (2015). Rapid assessment of marine non-native species in northern Scotland and a synthesis of existing Scottish records. *Aquatic Invasions*, *10*(1), 107–121.

Nasto, I., Cardone, F., Mastrototaro, F., Panetta, P., Rosso, A., Sanfilippo, R., … Tursi, A. (2018). Benthic invertebrates associated with subfossil cold-water coral frames and hardgrounds in the Albanian deep waters (Adriatic Sea). *Turkish Journal of Zoology*, *42*(4), 360–371. https://doi.org/10.3906/zoo-1708-44

Nekliudova, U. A., Schwaha, T. F., Kotenko, O. N., Gruber, D., Cyran, N., & Ostrovsky, A. N. (2019). Sexual reproduction of the placental brooder *Celleporella Hyalina* (Bryozoa, Cheilostomata) in the White Sea. *Journal of Morphology*, *280*(2), 278–299. https://doi.org/10.1002/jmor.20943

Nesnidal, M. P., Helmkampf, M., Bruchhaus, I., & Hausdorf, B. (2011). The complete mitochondrial genome of *Flustra foliacea* (Ectoprocta, Cheilostomata)-compositional bias affects phylogenetic analyses of lophotrochozoan relationships. *BMC Genomics*, *12*(1), 572.

Newcomer, K., Marraffini, M. L., & Chang, A. L. (2018). Distribution patterns of the introduced encrusting bryozoan Conopeum chesapeakensis (Osburn 1944; Banta et al. 1995) in an estuarine environment in upper San Francisco Bay. *Journal of Experimental Marine Biology and Ecology*, *504*, 20–31. https://doi.org/10.1016/j.jembe.2018.04.001

Nikulina, E. A. (2007). *Einhornia*, a new genus for electrids formerly classified as the *Electra crustulenta* species group (Bryozoa, Cheilostomata). *Schriften Des Naturwissenschaftlichen Vereins Für Schleswig-Holstein*, *69*, 29–40.

Nikulina, E. A. (2008). Electra scuticifera sp. Nov.: redescription of Electra pilosa from New Zealand as a new species (Bryozoa, Cheilostomata). *Schriften Des Naturwissenschaftlichen Vereins Für Schleswig-Holstein*, *70*, 91–98.

Nikulina, E. A., De Blauwe, H., & Reverter-Gil, O. (2012a). Molecular phylogenetic analysis confirms the species status of Electra verticillata (Ellis and Solander, 1786). In A. Ernst, P. Schäfer, & J. Scholz (Eds.), *Bryozoan Studies 2010* (Vol. 143, pp. 217–236). Berlin: Springer.

Nikulina, E. A., Ostrovsky, A. N., & Claereboudt, M. (2012b). A new species of the genus *Electra* (Bryozoa, Cheilostomata) from southern Oman, Arabian Sea. In A. Ernst, P. Schäfer, & J. Scholz (Eds.), *Bryozoan Studies 2010* (Vol. 143, pp. 203–216). Berlin: Springer.

Nikulina, E. A., & Schäfer, P. (2006). Bryozoans of the Baltic Sea. *Meyniana*, *58*, 75–95.

Nikulina, E. N., Hanel, R., & Schäfer, P. (2007). Cryptic speciation and paraphyly in the cosmopolitan bryozoan Electra pilosa - impact of the Tethys closing on species evolution. *Molecular Phylogenetics and Evolution*, *45*, 765–776.

Nordgaard, O. (1905). Hydrographical and biological investigations in Norwegian fiords. *Bergens Museums Meereskrifter*, *2*, 155–254.

Norman, A. M. (1868). Notes on some rare British Polyzoa, with descriptions of new species. *Quarterly Journal of Microscopical Science*, *8*(32), 212–222.

Norman, A. M. (1903). Notes on the natural history of East Finmark. Polyzoa. *Annals and Magazine of Natural History, Ser. 7*, *12*, 87–128.

Novosel, M. (2005). Bryozoans of the Adriatic Sea. *Denisia*, *16*, 231–246.

Novosel, M., & Pozar-Domac, A. (2001). Checklist of Bryozoa of the eastern Adriatic Sea. *Natura Croatica*, *10*(4), 367–421.

Novosel, M., Pozar-Domac, A., & Pasaric, M. (2004). Diversity and distribution of the Bryozoa along underwater cliffs in the Adriatic Sea with special reference to thermal regime. *Marine Ecology*, *25*(2), 155–170.

Nowak, M., Kuklinski, P., & Sicinski, J. (2014). Small-scale biomass variability of Antarctic bryozoans. *Studi Trentini Di Scienze Naturali*, *94*, 181–188.

O'Dea, A. (2006). Asexual propagation in the marine bryozoan *Cupuladria exfragminis*. *Journal of Experimental Marine Biology and Ecology*, *335*(2), 312–322.

O'Dea, A., Herrera-Cubilla, A., Fortunato, H., & Jackson, J. B. C. (2004). Life history variation in cupuladriid bryozoans from either side of the Isthmus of Panama. *Marine Ecology-Progress Series*, *280*, 145–161.

O'Donoghue, C. H. (1924). The Bryozoa (Polyzoa) collected by the S.S. 'Pickle'. *Report No. 3 of the Fisheries and Marine Biological Survey. For the Year 1922. Special Reports*, *10*, 1–63.

O'Donoghue, C. H. (1957). Some South African Bryozoa. *Transactions of the Royal Society of South Africa*, *35*, 71–95.

O'Donoghue, C. H., & de Watteville, D. (1939). The fishery grounds near Alexandria. XX. Bryozoa. *Fouad I Institute of Hydrobiology & Fisheries, Notes and Memoirs*, *34*, 1–58.

O'Donoghue, C. H., & de Watteville, D. (1944). Additional notes on Bryozoa from South Africa. *Annals of the Natal Museum*, *10*, 407–432.

Okamura, B. (1988). The influence of neighbors on the feeding of an epifaunal bryozoan. *Journal of Experimental Marine Biology and Ecology*, *120*(2), 105–123. https://doi.org/10.1016/0022-0981(88)90083-4

Oliver, J.-C., & Florence, W. K. (2016). A new species of *Taylorius* (Bryozoa: Escharinidae) from the east coast of South Africa. *African Natural History*, *12*, 1–4.

Orr, R. J. S., Waeschenbach, A., Enevoldsen, E. L. G., Boeve, J. P., Haugen, M. N., Voje, K. L., … Liow, L. H. (2019). Bryozoan genera *Fenestrulina* and *Microporella* no longer confamilial; multi-gene phylogeny supports separation. *Zoological Journal of the Linnean Society*, *186*(1), 190–199. https://doi.org/10.1093/zoolinnean/zly055

Osburn, R. C. (1912). Bryozoa from Labrador, Newfoundland and Nova Scotia collected by Dr Owen Bryant. *United States National Museum Proceedings*, *43*, 275–289.

Osburn, R. C. (1923). Part D. Bryozoa. In *Report of the Canadian Arctic expedition, 1913-18* (Vol. 8). Ottawa: F. A. Acland.

Osburn, R. C. (1927). The Bryozoa of Curacao. *Bijdragen Tot de Dierkunde*, *25*, 123–132.

Osburn, R. C. (1933). Bryozoa of the Mount Desert Region. In W. Procter (Ed.), *Biological Survey of the Mount Desert region* (pp. 291–385). Philadelphia: Wistar Institute of Anatomy and Biology.

Osburn, R. C. (1936). Bryozoa collected in the American Arctic by Captain R.A. Bartlett. *Journal of the Washington Academy of Science*, *26*, 538–543.

Osburn, R. C. (1940). Bryozoa of Porto Rico with a résumé of the West Indian bryozoan fauna. *Scientific Survey of Porto Rico and the Virgin Islands*, *16*, 321–486.

Osburn, R. C. (1944). A survey of the Bryozoa of Chesapeake Bay. *Chesapeake Biological Laboratory Publication*, *63*, 3–55.

Osburn, R. C. (1947). Bryozoa of the Allan Hancock Atlantic Expedition, 1939. *Allan Hancock Atlantic Expedition Report*, *5*, 1–66.

Osburn, R. C. (1950). *Bryozoa of the Pacific coast of America, Part 1, Cheilostomata-Anasca.* Los Angeles: University of Southern California Press.

Ostrovsky, A. N. (1998). Comparative studies of ovicell anatomy and reproductive patterns in *Cribrilina annulata* and *Celleporella hyalina* (Bryozoa: Cheilostomatida). *Acta Zoologica*, *79*(4), 287–318.

Ostrovsky, A. N. (2008). The parental care in cheilostome bryozoans: A historical review. In P. N. Wyse Jackson & M. E. Spencer Jones (Eds.), *Annals of Bryozoology 2: Aspects of the history of research on bryozoans* (Vol. 2, pp. 211–245). Dublin: International Bryozoology Association.

Ostrovsky, A. N., Cáceres-Chamizo, J. P., Vávra, N., & Berning, B. (2011). Bryozoa of the Red Sea: History and current state of research. In P. N. Wyse Jackson & M. E. Spencer Jones (Eds.), *Annals of Bryozoology 3: Aspects of the History of Research on Bryozoans* (pp. 67–97). Dublin: International Bryozoology Association.

Ostrovsky, A. N., Vávra, N., & Porter, J. S. (2008). Sexual reproduction in gymnolaemate Bryozoa: History and perspectives of the research. In P. N. Wyse Jackson & M. E. Spencer Jones (Eds.), *Annals of Bryozoology 2: Aspects of the history of research on bryozoans.* Dublin: International Bryozoology Association.

Pagès-Escolà, M., Hereu, B., Garrabou, J., Montero-Serra, I., Gori, A., Gómez-Gras, D., … Linares, C. (2018). Divergent responses to warming of two common co-occurring Mediterranean bryozoans. *Scientific Reports*, *8*(1). https://doi.org/10.1038/s41598-018-36094-9

Peters, L., Konig, G. M., Terlau, H., & Wright, A. D. (2002). Four new bromotryptamine derivatives from the marine bryozoan *Flustra foliacea. Journal of Natural Products*, *65*(11), 1633–1637.

Pizzaferri, C. (2006). A new subspecies of Cupuladria cavernosa Cadée from the Pliocene of western Emilia (N. Italy) (Bryozoa Gymnolaemata Cheilostomatida Cupuladriidae). *Quaderno Di Studi E Notizie Di Storia Naturale Della Romagna*, *22*, 39–51.

Porter, J. S., Nunn, J. D., Ryland, J. S., Minchin, D., & Jones, M. E. S. (2017). The status of non-native bryozoans on the north coast of Ireland. *Bioinvasions Records*, *6*(4), 321–330.

Porter, J. S., Spencer Jones, M. E., Kuklinski, P., & Rouse, S. (2015). First records of marine invasive non-native Bryozoa in Norwegian coastal waters from Bergen to Trondheim. *BioInvasions Records*, *4*(3), 157–169. https://doi.org/doi:%20http://dx.doi.org/10.3391/bir.2015.4.3.02

Powell, N. A. (1968). Bryozoa (Polyzoa) of Arctic Canada. *Journal of the Fisheries Research Board of Canada*, *25*, 2269–2320.

Powell, N. A. (1968). Studies on Bryozoa (Polyzoa) of the Bay of Fundy region. II. - Bryozoa from fifty fathoms, Bay of Fundy (I). *Cahiers de Biologie Marine*, *9*, 247–259.

Powell, N. A. (1969). A checklist of Indo-Pacific Bryozoa in the Red Sea. *Israel Journal of Zoology*, *18*, 357–362.

Powell, N. A., & Crowell, G. D. (1967). Studies on Bryozoa (Polyzoa) of the Bay of Fundy Region. 1. Bryozoa of the intertidal zone of the Minas Basin and Bay of Fundy. *Cahiers de Biologie Marine*, *8*, 331–347.

Quaiyum, S., Fortunato, H., Gonzaga, L., & Okabe, S. (2018). Antimicrobial Activity in the Marine Cheilostome Bryozoan *Cryptosula* zavjalovensis Kubanin, 1976. *Journal of Antimicrobial Agents*, *04*(03). https://doi.org/10.4172/2472-1212.1000178

Rabaoui, L., Tlig-Zouari, S., Cosentino, A., & Ben Hassine, O. K. (2009). Associated fauna of the fan shell *Pinna nobilis* (Mollusca: Bivalvia) in the northern and eastern Tunisian coasts. *Scientia Marina*, *73*(1), 129–141.

Ramalho, L. V., & Calliari, L. (2015). Bryozoans from Rio Grande do Sul Continental Shelf, Southern Brazil. *Zootaxa*, *3955*(4), 569–587.

Ramalho, L. V., López-Fé, C. M., & Rueda, J. L. (2018). Three species of *Reteporella* (Bryozoa: Cheilostomata) in a diapiric and mud volcano field of the Gulf of Cádiz, with the description of *Reteporella victori* n. Sp. *Zootaxa*, *4375*(1), 90. https://doi.org/10.11646/zootaxa.4375.1.4

Ramalho, L. V., Muricy, G., & Taylor, P. D. (2005). Taxonomy and distribution of *Bugula* (Bryozoa: Cheilostomata; Anasca) in Rio de Janeiro State, Brasil. In H. I. Moyano, J. M. Cancino, & P. N. Wyse Jackson (Eds.), *Bryozoan Studies 2004* (pp. 231–243). Leiden: Balkema.

Ramalho, L. V., Muricy, G., & Taylor, P. D. (2008). Taxonomy of *Beania* Johnston, 1840 (Bryozoa, Flustrina) from Arraial do Cabo, Rio de Janeiro State, Brazil. *Arquivos Do Museu Nacional, Rio de Janeiro*, *66*(3-4), 499–508.

Ramalho, L. V., Taylor, P. D., Moraes, F. C., Moura, R., Amado-Filho, G. M., & Bastos, A. C. (2018). Bryozoan framework composition in the oddly shaped reefs from Abrolhos Bank, Brazil, southwestern Atlantic: Taxonomy and ecology. *Zootaxa*, *4483*(1), 155–186. https://doi.org/10.11646/zootaxa.4483.1.6

Ramalho, L. V., Taylor, P. D., & Muricy, G. (2014). New records of *Catenicella* de Blainville, 1830 (Catenicellidae: Cheilostomata: Ascophora) in Rio de Janeiro State, Brazil. *Check List*, *10*(1), 170–174.

Rao, K., & Ganapati, P. N. (1978). Ecology of fouling bryozoans at Visakhapatnam Harbour. *Proceedings of the Indian Academy of Sciences Section B*, *87*(3), 63–75.

Reverter-Gil, O., Berning, B., & Souto, J. (2015a). Diversity and systematics of *Schizomavella* species (Bryozoa: Bitectiporidae) from the bathyal NE Atlantic. *PLoS ONE*, *10*(10), e0139084. https://doi.org/doi:10.1371/journal.pone.0139084

Reverter Gil, O., & Fernández Pulpeiro, E. (1999a). Some little-known species of Bryozoa described by J. Jullien. *Journal of Natural History*, *33*(9), 1403–1418.

Reverter-Gil, O., & Fernández-Pulpeiro, E. (1997). Two new species of *Schizomavella* (Bryozoa, Cheilostomatida). *Cahiers de Biologie Marine*, *38*, 1–6.

Reverter Gil, O., & Fernández Pulpeiro, E. (1999b). Some records of bryozoans from NW Spain. *Cahiers de Biologie Marine*, *40*(1), 35–45.

Reverter-Gil, O., & Souto, J. (2015). Redescription of some species of Bryozoa described by J. Jullien and L. Calvet in the NE Atlantic. *European Journal of Taxonomy*, *157*, 1–17.

Reverter-Gil, O., Souto, J., & Fernandez-Pulpeiro, E. (2011). Revision of the genus *Crepis* Jullien (Bryozoa: Cheilostomata) with description of a new genus and family and notes on Chlidoniidae. *Zootaxa*, *2993*, 1–22.

Reverter-Gil, O., Souto, J., & Fernández-Pulpeiro, E. (2012a). A new genus of Lanceoporidae (Bryozoa, Cheilostomata). *Zootaxa*, *3339*, 1–29.

Reverter-Gil, O., Souto, J., & Fernández Pulpeiro, E. (2009). Three new species of Iberian cheilostomate Bryozoa. *Journal of the Marine Biological Association of the United Kingdom*, *89*(7), 1499–1506. https://doi.org/10.1017/S0025315409000496

Reverter-Gil, O., Souto, J., & Fernández-Pulpeiro, E. (2012b). New and little known species of Bryozoa from Iberian Atlantic waters. *Zoosystema*, *34*(1), 157–170.

Reverter-Gil, O., Souto, J., Novosel, M., & Tilbrook, K. J. (2015b). Adriatic species of *Schizomavella* (Bryozoa: Cheilostomata). *Journal of Natural History*, *50*(5-6), 281–321.

Reverter-Gil, O., Souto, J., & Trigo, J. E. (2019). New species and new records of bryozoans from Galicia (NW Spain). *Journal of Natural History*, *53*(3-4), 221–251. https://doi.org/10.1080/00222933.2019.1582815

Ridley, S. O. (1881). Account of Polyzoa collected during the survey of H.M.S. 'Alert,' in the Straits of Magellan and on the Coast of Patagonia. *Proceedings of the Zoological Society of London*, 44–61.

Riisgard, H. U., & Manriquez, P. (1997). Filter-feeding in fifteen marine ectoprocts (Bryozoa): Particle capture and water pumping. *Marine Ecology Progress Series*, *154*, 223–239. https://doi.org/10.3354/meps154223

Robertson, A. (1905). Non-incrusting cheilostomatous Bryozoa of the west coast of North America. *University of California Publications in Zoology.*

Robertson, A. (1908). The incrusting chilostomatous Bryozoa of the west coast of North America. *University of California Publications in Zoology*, *4*(5), 253–344.

Rodgers, P. J., & Woollacott, R. M. (2006). Systematics, variation, and developmental instability: Analysis of spine patterns in ancestrulae of a common bryozoan. *Journal of Natural History*, *40*(21-22), 1351–1368. Retrieved from://000241266500006

Rogick, M. D. (1956). Bryozoa of the United States Navy's 1947-1948 Antarctic Expedition, I-IV. *Proceedings of the United States National Museum*, *105*(3358), 221–317.

Ross, J. R. P. (1974). Reef associated Ectoprocta from central region, Great Barrier Reef. *Proceedings of the Second International Coral Reef Symposium*, *1*, 349–352.

Rosso, A., Di Martino, E., Sanfilippo, R., & Di Martino, V. (2012). Bryozoan communities and thanatocoenoses from submarine caves in the Plemmirio Marine Protected Area (SE Sicily). In A. Ernst, P. Schäfer, & J. Scholz (Eds.), *Bryozoan Studies 2010* (Vol. 143, pp. 251–269). Berlin: Springer.

Rosso, A., Gerovasileiou, V., Sanfilippo, R., & Guido, A. (2019). Undisclosed bryodiversity of submarine caves of the Aegean Sea (Eastern Mediterranean). *Proceedings of the 2nd Mediterranean Symposium on the Conservation of Dark Habitats*, 47–52. Antalya, Turkey.

Rosso, A., Sciuto, F., Sanfilippo, R., & Jones, M. S. (2017). The bryozoan genus *Arbocuspis* (Cheilostomata, Electridae) from the Indian Ocean, with description of a new species from off southwestern Thailand, Andaman Sea. *Zootaxa*, *4282*(1), 95–110.

Rosso, A., & Taylor, P. D. (2002). A new anascan cheilostome bryozoan from Icelandic deep waters and its uniserial colony growth pattern. *Sarsia*, *87*, 35–46.

Rouse, S., Spencer Jones, M. E., & Porter, J. S. (2013). Spatial and temporal patterns of bryozoan distribution and diversity in the Scottish sea regions. *Marine Ecology*, *35*(s1), 85–102.

Rörig, L. R., Ottonelli, M., Itokazu, A. G., Maraschin, M., Lins, J. V. H., Abreu, P. C. V., … Ramalho, L. V. (2017). Blooms of bryozoans and epibenthic diatoms in an urbanized sandy Beach (Balneário Camboriú-SC-Brazil): Dynamics, possible causes and biomass characterization. *Brazilian Journal of Oceanography*, *65*(4), 678–694.

Rubin, J. A. (1985). Mortality and avoidance of competitive overgrowth in encrusting Bryozoa. *Marine Ecology Progress Series*, *23*, 291–299.

Russ, G. R. (1982). Overgrowth in a marine epifaunal community: Competitive hierarchies and competitive networks. *Oecologia*, *53*, 12–19.

Ryland, J. S. (1969). A nomenclatural index to "A History of the British Marine Polyzoa" by T. Hincks (1880). *Bulletin of the British Museum (Natural History), Zoology*, *17*(6), 205–260.

Ryland, J. S. (1995). Bryozoa. In P. J. Hayward & J. S. Ryland (Eds.), *Handbook of the marine fauna of north-west Europe* (pp. 629–661). Oxford, New York: Oxford University Press.

Ryland, J. S., Bishop, J. D., De Blauwe, H., El Nagar, A., Minchin, D., Wood, C. A., & Yunnie, A. L. (2011). Alien species of *Bugula* (Bryozoa) along the Atlantic coasts of Europe. *Aquatic Invasions*, *6*(1), 17–31.

Ryland, J. S., & Gordon, D. P. (1977). Some New Zealand and British species of *Hippothoa* (Bryozoa: Cheilostomata). *Journal of the Royal Society of New Zealand*, *7*(1), 17–49.

Ryland, J. S., & Hayward, P. (1992). Bryozoa from Heron Island, Great Barrier Reef. *Memoirs of the Queensland Museum*, *32*(1), 223–301.

Ryland, J. S., Holt, R., Loxton, J., Spencer Jones, M. E., & Porter, J. S. (2014). First occurrence of the non-native bryozoan *Schizoporella japonica* Ortmann (1890) in Western Europe. *Zootaxa*, *3780*(3), 481–502.

Ryland, J. S., & Stebbing, A. R. D. (1971). Settlement and orientated growth in epiphytic and epizoic bryozoans. *Fourth European Marine Biology Symposium*, 105–124.

Ryland, J. S., & Stebbing, A. R. D. (1971). Two little known bryozoans from the west of Ireland. *The Irish Naturalist's Journal*, *17*(3), 65–70.

Santagata, S. (2015). Ectoprocta. In A. Wanninger (Ed.), *Evolutionary Developmental Biology of Invertebrates 2 Lophotrochozoa (Spiralia)* (pp. 247–262). Wien: Springer. Retrieved from http://link.springer.com/chapter/10.1007/978-3-7091-1871-9_11

Santagata, S., Ade, V., Mahon, A. R., Wisocki, P. A., & Halanych, K. M. (2018). Compositional differences in the habitat-forming bryozoan communities of the antarctic shelf. *Frontiers in Ecology and Evolution*, *6*. https://doi.org/10.3389/fevo.2018.00116

Santana, F. T., Ramalho, L. V., & Gumarães, C. P. (2009). A new species of *Metrarabdotos* (Bryozoa, Ascophora) from Brazil. *Zootaxa*, *2222*(57-65).

Schäfer, P. (2008). Diversity patterns of modern Arctic and Antarctic bryozoans. In H. Okada, S. F. Mawatari, N. Suzuki, & P. Gautam (Eds.), *Origin and Evolution of Natural Diversity* (pp. 57–66).

Schäfer, P., Fortunato, H., & Blaschek, H. (2014). Calcification patterns in the cheilostome bryozoan Flustra foliacea (L.) From the North Sea and Baltic Sea. *Studi Trentini Di Scienze Naturali*, *94*, 213–221.

Scholz, J., Mawatari, S. F., & Hirose, M. (2008). The Bryozoan diversity mystery: Why do we have about 1000 Species in Japanese waters? *Origin and Evolution of Natural Diversity : Proceedings of the International Symposium, the Origin and Evolution of Natural Diversity, Held from 1-5 October 2007 in Sapporo, Japan*, 129–135.

Schopf, T. J. (1973). Ergonomics of polymorphism: Its relation to the colony as the unit of natural selection in species of the phylum Ectoprocta. In R. S. Boardman, A. H. Cheetham, & W. A. Oliver (Eds.), *Animal Colonies: Development and Function Through Time* (pp. 247–294).

Schopf, T. J. M. (1969). Paleoecology of ectoprocts (bryozoans). *Journal of Paleontology*, *43*(2), 234–244.

Schopf, T. J. M. (1974). Ectoprocts as associates of coral reefs: St. Croix, U.S. Virgin Islands. *Proceedings of the Second International Reef Symposium*, *1*, 353–356. Brisbane: Great Barrier Reef Committee.

Sears, M. A. B., & Woollacott, R. M. (2008). Alice Robertson: Educator and marine zoologist. In P. N. Wyse Jackson & M. E. Spencer Jones (Eds.), *Annals of Bryozoology 2:*

Aspects of the history of research on bryozoans. (pp. 305–345). Dublin: International Bryozoology Association.

Sears, M. A., & Woollacott, R. M. (2011). Reverend William F. Lynch: A Life in Science and Education. In *Annals of Bryozoology 3: Aspects of the history of research on bryozoans.*

Sears, M. A., & Woollacott, R. M. (2014). *Benjamin Harrison Grave: American Marine Invertebrate Zoologist.*

Sebastian, P., & Cumming, R. L. (2016). Three new species of *Calyptotheca* (Bryozoa: Lanceoporidae) from the great barrier reef, tropical Australia. *Zootaxa*, *4079*(4), 467–479.

Seo, J. E. (1994). Two species of Celleporaria (Cheilostomata: Bryozoa) from Korea. *Korean Journal of Systematic Zoology*, *10*(2), 189–197.

Seo, J. E. (1996). Two new species of Membraniporoidea (Bryozoa: Cheilostomata) from Korea. *Korean Journal of Systematic Zoology*, *12*(1), 45–51.

Seo, J. E., & Gong, Y. H. (2006). A new species and two new records of cheilostomata (Bryozoa) from Korea. *Korean Journal of Systematic Zoology*, *22*(1), 13–16.

Seo, J. E., & Min, B. S. (2009). A faunistic study on cheilostomatous bryozoans from the shoreline of South Korea, with two new species. *Korean Journal of Systematic Zoology*, *25*(1), 19–40.

Seroy, S., & Grünbaum, D. (2018). Individual and population level effects of ocean acidification on a predator-prey system with inducible defenses: Bryozoan-nudibranch interactions in the Salish Sea. *Marine Ecology Progress Series*, *607*, 1–18. https://doi.org/10.3354/meps12793

Sharp, K. H., Davidson, S. K., & Haygood, M. G. (2007). Localization of 'Candidatus Endobugula sertula'and the bryostatins throughout the life cycle of the bryozoan Bugula neritina. *The ISME Journal*, *1*(8), 693–702.

Shier, D. E. (1964). Marine Bryozoa from northwest Florida. *Bulletin of Marine Science*, *14*(4), 603–662.

Silén, L. (1950). On the Mobility of Entire Zoids in Bryozoa. *Acta Zoologica*, *31*(2-3), 349–386. https://doi.org/10.1111/j.1463-6395.1950.tb00515.x

Skinner, L. F. (2014). Foraminifera as predators on encrusting bryozoans. *Journal of Foraminiferal Research*, *44*(1), 58–61.

Smith, A. M. (2014). Growth and Calcification of Marine Bryozoans in a Changing Ocean. *The Biological Bulletin*, *226*(3), 203–210. Retrieved from http://www.biolbull.org/content/226/3/203

Smith, A. M., & Girvan, E. (2010). Understanding a bimineralic bryozoan: Skeletal structure and carbonate mineralogy of *Odontionella cyclops* (Foveolariidae: Cheilostomata: Bryozoa) in New Zealand. *Palaeogeography, Palaeoclimatology, Palaeoecology*, *289*, 113–122. https://doi.org/doi:10.1016/j.palaeo.2010.02.022

Smith, A. M., & Gordon, D. P. (2011). Bryozoans of southern New Zealand: A field identification guide. *New Zealand Aquatic Environment and Biodiversity Report*, *75*, 1–64.

Smith, A. M., & Lawton, E. I. (2010). Growing up in the temperate zone: Age, growth, calcification and carbonate mineralogy of Melicerita chathamensis (Bryozoa) in southern New Zealand. *Palaeogeography, Palaeoclimatology, Palaeoecology*, *298*, 271–277. https://doi.org/doi:10.1016/j.palaeo.2010.09.033

Smith, A. M., Spencer Jones, M. E., & Wyse Jackson, P. N. (2014). Bryozoans of the Krusenstern Expedition (1803-1806). *International Bryozoology Association, Dublin*, 183–194.

Sokolover, N., Ostrovsky, A. N., & Ilan, M. (2018). *Schizoporella errata* (Bryozoa, Cheilostomata) in the Mediterranean Sea: Abundance, growth rate, and reproductive strategy. *Marine Biology Research*, *14*(8), 868–882. https://doi.org/10.1080/17451000.2018.1526385

Sokolover, N., Taylor, P. D., & Ilan, M. (2016). Bryozoa from the Mediterranean coast of Israel. *Mediterranean Marine Science*, *17*(2), 440–458. https://doi.org/DOI:%20http://dx.doi.org/10.12681/mms.1390

Sosa-Yañez, A., Fernández, L. H., & Olivera, Y. (2014). Primer registro de Adeonellopsis subsulcata (Smitt, 1873)(Bryozoa Gymnolaemata) en Cuba. *Revista Ciencias Marinas Y Costeras*, *6*(1), 29–36.

Sosa-Yañez, A., Vieira, L. M., & Solís-Marín, F. A. (2015). A new cheilostome bryozoan genus, *Abditoporella* (Hippoporidridae), from the eastern Pacific. *Zootaxa*, *3994*(2), 275–282.

Soule, D. F., & Soule, J. D. (1975). Species groups in Watersiporidae. In *Documents Des Laboratoires de Géologie de La Faculté Des Sciences de Lyon*: *Vol. 3. Bryozoa 1974* (pp. 299–309). Lyon: Université Claude Bernard.

Soule, J. D. (1959). Results of the Puritan-American Museum of Natural History Expedition to western Mexico. 6. Anascan Cheilostomata (Bryozoa) of the Gulf of California. *American Museum Novitates*, *1969*, 1–54.

Soule, J. D. (1961). Results of the Puritan-American Museum of Natural History Expedition to western Mexico. 13. Ascophoran Cheilostomata (Bryozoa) of the Gulf of California. *American Museum Novitates*, *2053*, 1–66.

Soule, J. D., Soule, D. F., & Chaney, H. W. (1998). Two new tropical Pacific species of *Cribralaria* (Bryozoa: Cribrilinidae) and a review of known species. *Irene Mcculloch Foundation Monograph Series*, *3*, 1–23.

Souto, J. (2019). Secondary homonymy in Bryozoa: The case of *Reteporella jullieni* (Cheilostomatida). *Zootaxa*, *4565*(2), 292. https://doi.org/10.11646/zootaxa.4565.2.13

Souto, J., Berning, B., & Ostrovsky, A. N. (2016). Systematics and diversity of deep-water Cheilostomata (Bryozoa) from Galicia Bank (NE Atlantic). *Zootaxa*, *4067*(4), 401–459.

Souto, J., Kaufmann, M. J., & Canning-Clode, J. (2015a). New species and new records of bryozoans from shallow waters of Madeira Island. *Zootaxa*, *3925*(4), 581–593.

Souto, J., Nascimento, K. B., Reverter-Gil, O., & Vieira, L. M. (2019). Dismantling the *Beania magellanica* (Busk, 1852) species complex (Bryozoa, Cheilostomata): Two new species from European waters. *Marine Biodiversity*, *49*(3), 1505–1518. https://doi.org/10.1007/s12526-018-0925-2

Souto, J., Ramalhosa, P., & Canning-Clode, J. (2015). Three non-indigenous species from Madeira harbors, including a new species of Parasmittina (Bryozoa). *Marine Biodiversity*.

Souto, J., & Reverter-Gil, O. (2019). Identity of bryozoan species described by Jullien & Calvet from the Bay of Biscay historically attributed to Smittia. *Zootaxa*, *4545*(1), 105. https://doi.org/10.11646/zootaxa.4545.1.6

Souto, J., Reverter-Gil, O., & de Blauwe, H. (2015b). New and little known species of *Celleporina* Gray, 1848 (Bryozoa, Cheilostomata) from the Atlantic–Mediterranean region. *Journal of the Marine Biological Association of the United Kingdom*, *95*(4), 723–734.

Souto, J., Reverter-Gil, O., De Blauwe, H., & Fernández Pulpeiro, E. (2014a). New records of bryozoans from Portugal. *Cahiers de Biologie Marine*, *55*, 129–150.

Souto, J., Reverter-Gil, O., & Fernandez-Pulpeiro, E. (2010). Bryozoa from detritic bottoms in the Menorca Channel (Balearic Islands, western Mediterranean), with notes on the genus Cribellopora. *Zootaxa*, *2536*(1), 36–52.

Souto, J., Reverter-Gil, O., & Fernández-Pulpeiro, E. (2010). Gymnolaemate bryozoans from the Algarve (southern Portugal): New species and biogeographical considerations. *Journal of the Marine Biological Association of the United Kingdom*, *90*, 1417–1439. Retrieved from http://dx.doi.org/10.1017/S0025315409991640

Souto, J., Reverter-Gil, O., & Fernández-Pulpeiro, E. (2011). Redescription of some bryozoan species originally described by J. Jullien from Iberian waters. *Zootaxa*, *2827*, 31–53.

Souto, J., Reverter-Gil, O., & Ostrovsky, A. N. (2014b). New species of Bryozoa from Madeira associated with rhodoliths. *Zootaxa*, *3795*(2), 135–151. https://doi.org/http://dx.doi.org/10.11646/zootaxa.3795.2.3

Stebbing, A. R. D. (1971). The epizoic fauna of *Flustra foliacea* (Bryozoa). *Journal of the Marine Biological Association of the United Kingdom*, *51*, 283–300.

Stricker, S. A. (1989). Settlement and metamorphosis of the marine bryozoan *Membranipora membranacea*. *Bulletin of Marine Science*, *45*(2), 387–405.

Ström, R. (1977). Brooding patterns of bryozoans. In R. M. Woollacott & R. L. Zimmer (Eds.), *Biology of bryozoans* (pp. 23–55). Academic Press.

Sutherland, J. P. (1981). The fouling community at Beaufort, North Carolina: A study in stability. *The American Naturalist*, *118*, 499–519.

Sutherland, J. P., & Karlson, R. H. (1977). Development and stability of the fouling community at Beaufort, North Carolina. *Ecological Monographs*, *47*, 425–446.

Suwa, T., Dick, M. H., & Mawatari, S. F. (1998). A new species of *Microporella* (Bryozoa, Cheilostomata) from Alaska. *Zoological Science*, *15*, 589–592.

Suwa, T., & Mawatari, S. F. (1998). Revision of seven species of *Microporella* (Bryozoa, Cheilostomatida) from Hokkaido, Japan, using new taxonomic characters. *Journal of Natural History*, *32*, 895–922.

Tadesse, M., Tabudravu, J. N., Jaspars, M., Strøm, M. B., Hansen, E., Andersen, J. H., … Haug, T. (2011). The antibacterial ent-eusynstyelamide B and eusynstyelamides D, E, and F from the Arctic bryozoan *Tegella cf. spitzbergensis*. *Journal of Natural Products*, *74*(4), 837–841.

Taylor, P. D., & Monks, N. (1997). A new cheilostome bryozoan genus pseudoplanktonic on molluscs and algae. *Invertebrate Biology*, *116*(1), 39–51.

Taylor, P. D., & Tan, S.-H. A. (2015). Cheilostome Bryozoa from Penang and Langkawi, Malaysia. *European Journal of Taxonomy*, *149*, 1–34.

Taylor, P. D., Tan S.-H., A., Kudryavstev, A. B., & Schopf, J. W. (2016). Carbonate mineralogy of a tropical bryozoan biota and its vulnerability to ocean acidification. *Marine Biology Research*, *12*(7), 776–780.

Teixidó, N., Garrabou, J., Gutt, J., & Arntz, W. E. (2004). Recovery in Antarctic benthos after iceberg disturbance: Trends in benthic composition, abundance and growth forms. *Marine Ecology Progress Series*, *278*, 1–16.

Temkin, M. H. (1994). Gamete spawning and fertilization in the Gymnolaemate Bryozoan *Membranipora membranacea. The Biological Bulletin*, *187*(2), 143–155.

Temkin, M. H. (1996). Comparative fertilization biology of gymnolaemate bryozoans. *Marine Biology*, *127*(2), 329–339.

Temkin, M. H., & Bortolami, S. B. (2004). Waveform dynamics of spermatozeugmata during the transfer from paternal to maternal individuals of *Membranipora membranacea. The Biological Bulletin*, *206*(1), 35–45.

Tian, X.-R., Tang, H.-F., Li, Y.-S., Lin, H.-W., Chen, X.-L., Ma, N., … Zhang, P.-H. (2011). New cytotoxic oxygenated sterols from the marine bryozoan *Cryptosula pallasiana*. *Marine Drugs*, *9*(2), 162–183.

Tilbrook, K. J. (1999). Description of Hippopodina feegeensis and three other species of Hippopodina Levinsen, 1909 (Bryozoa: Cheilostomatida). *Journal of Zoology, London*, *247*, 449–456.

Tilbrook, K. J. (2000). New bryozoan family from the Indo-Pacific shows unexpected diversity. *Journal of Marine Biological Association of the United Kingdom*, *80*, 1129–1130.

Tilbrook, K. J. (2011). New genus for a unique species of Indo-West Pacific bryozoan. *Zootaxa*, *3134*, 63–67.

Tilbrook, K. J. (2012a). Bryozoa, Cheilostomata: First records of two invasive species in Australia and the northerly range extension for a third. *Check List*, *8*(1), 181–183.

Tilbrook, K. J. (2012b). Review of the bryozoan genus Bryopesanser Tilbrook, 2006 (Escharinidae: Cheilostomata) with the description of 11 new species. *Zootaxa*, *3165*, 39–63.

Tilbrook, K. J., & Cook, P. L. (2005). Petraliellidae Harmer, 1957 (Bryozoa: Cheilostomata) from Queensland, Australia. *Systematics and Biodiversity*, *2*(3), 319–339.

Tilbrook, K. J., & Gordon, D. P. (2015). Bryozoa from the Straits of Johor, Singapore, with the description of a new species. *Raffles Bulletin of Zoology*, *31*, 255–263.

Tilbrook, K. J., & Gordon, D. P. (2016). Checklist of Singaporean Bryozoa and Entoprocta. *Raffles Bulletin of Zoology.*

Tilbrook, K. J., & Grischenko, A. V. (2004). New sub-Arctic species of the tropical genus *Antropora* (Bryozoa: Cheilostomata): A gastropod-pagurid crab associate. *Journal of the Marine Biological Association of the United Kingdom*, *84*, 1001–1004.

Tilbrook, K. J., Hayward, P. J., & Gordon, D. P. (2001). Cheilostomatous Bryozoa from Vanuatu. *Zoological Journal of the Linnean Society*, *131*, 35–109.

Tilbrook, K. J., & Vieira, L. M. (2012). *Scrupocellaria* (Bryozoa: Cheilostomata) from the Queensland coast, with the description of three new species. *Zootaxa*, *3528*(1), 29–48.

Tzioumis, V. (1994). Bryozoan stolonal outgrowths: A role in competitive interactions? *Journal of the Marine Biological Association of the United Kingdom*, *74*(1), 203–210.

Vaughan, D. G., Barnes, D. K., Fretwell, P. T., & Bingham, R. G. (2011). Potential seaways across west Antarctica. *Geochemistry, Geophysics, Geosystems*, *12*(10).

Venkatraman, C., Rajan, R., Louis, S., Shrinivaasu, S., & Padmanaban, P. (2016). Bryozoans of Gulf of Mannar Marine Biosphere Reserve, Southeast Coast of India. *Records of the Zoological Survey of India*, *116*(2), 167–189.

Vieira, L. M., Almeida, A. C. S., & Winston, J. E. (2016). Taxonomy of intertidal cheilostome Bryozoa of Maceió, northeastern Brazil. Part 1: Suborders Inovicellina, Malacostegina and Thalamoporellina. *Zootaxa*, *4097*(1), 59–83. https://doi.org/http://doi.org/10.11646/zootaxa.4097.1.3

Vieira, L. M., Farrapeira, C. M. R., Amaral, F. D., & Lira, S. M. A. (2012). Bryozoan biodiversity in Saint Peter and Saint Paul Archipelago, Brazil. *Cahiers de Biologie Marine*, *53*, 159–167.

Vieira, L. M., & Gordon, D. P. (2010). Eutaleola, a replacement name for the homonym Euteleia (Bryozoa: Pasytheidae). *Zoologia*, *27*(4), 646–648.

Vieira, L. M., Gordon, D. P., & Correia, M. D. (2007). First record of a living ditaxiporine catenicellid in the Atlantic, with a description of *Vasignyella ovicellata* n. Sp. (Bryozoa). *Zootaxa*, *1582*, 49–58.

Vieira, L. M., Gordon, D. P., Souza, F. B. C., & Haddad, M. A. (2010a). New and little-known cheilostomatous Bryozoa from the south and southeastern Brazilian continental

shelf and slope. *Zootaxa*, *2722*, 1–53.

Vieira, L. M., & Migotto, A. E. (2015). *Membraniporopsis tubigera* (Osburn, 1940) (Bryozoa) on floating substrata: Evidence of a dispersal mechanism in the western Atlantic. *Marine Biodiversity*, *45*(2), 155–156.

Vieira, L. M., Migotto, A. E., & Winston, J. E. (2008). Synopsis and annotated checklist of Recent marine Bryozoa from Brazil. *Zootaxa*, *1810*, 1–39.

Vieira, L. M., Migotto, A. E., & Winston, J. E. (2010b). Marcusadorea, a new genus of lepralioid bryozoan from warm waters. *Zootaxa*, *2348*, 57–68.

Vieira, L. M., Migotto, A. E., & Winston, J. E. (2010c). Shallow-water species of *Beania* Johnston, 1840 (Bryozoa, Cheilostomata) from the tropical and subtropical Western Atlantic. *Zootaxa*, *2550*, 1–20.

Vieira, L. M., & Spencer Jones, M. E. (2012). The identity of Sertularia reptans Linnaeus, 1758 (Bryozoa, Candidae). *Zootaxa*, *3563*, 26–42.

Vieira, L. M., Spencer Jones, M. E., & Taylor, P. D. (2014a). The identity of the invasive fouling bryozoan Watersipora subtorquata (d'Orbigny) and some other congeneric species. *Zootaxa*, *3857*(2), 151–182.

Vieira, L. M., Spencer Jones, M. E., & Winston, J. E. (2013a). Cradoscrupocellaria, a new bryozoan genus for Scrupocellaria bertholletii (Audouin) and related species (Cheilostomata, Candidae): Taxonomy, biodiversity and distribution. *Zootaxa*, *3707*(1), 1–63. https://doi.org/http://dx.doi.org/10.11646/zootaxa.3707.1.1

Vieira, L. M., Spencer Jones, M. E., & Winston, J. E. (2013b). Resurrection of the genus *Licornia* for *Scrupocellaria jolloisii* (Bryozoa) and related species, with documentation of *L. jolloisii* as a non-indigenous species in the western Atlantic. *Journal of the Marine Biological Association of the United Kingdom*, *93*(7), 1911–1921. https://doi.org/10.1017/S0025315413000301

Vieira, L. M., Spencer Jones, M. E., Winston, J. E., Migotto, A. E., & Marques, A. C. (2014b). Evidence for polyphyly of the genus *Scrupocellaria* (Bryozoa: Candidae) based on a phylogenetic analysis of morphological characters. *PLoS ONE*, *9*(4), e95296. https://doi.org/10.1371/journal.pone.0095296

Vieira, L. M., & Stampar, S. N. (2014). A new *Fenestrulina* (Bryozoa, Cheilostomata) commensal with tube-dwelling anemones (Cnidaria, Ceriantharia) in the tropical southwestern Atlantic. *Zootaxa*, *3780*(2), 365–374.

Vieira, L. M., Winston, J. E., & Fehlauer-Ale, K. H. (2012). Nine New Species of *Bugula* Oken (Bryozoa: Cheilostomata) in Brazilian Shallow Waters. *PLoS ONE*, *7*(7), e40492. https://doi.org/10.1371/journal.pone.0040492

Vigeland, I. (1971). Port Phillip Bay Survey 2. Bryozoa. *Memoirs of the National Museum of Victoria*, *32*, 65–81.

Voronkov, A., Hop, H., & Gulliksen, B. (2016). Zoobenthic communities on hard-bottom habitats in Kongsfjorden, Svalbard. *Polar Biology*, *39*(11), 2077–2095.

Waeschenbach, A., Porter, J. S., & Hughes, R. N. (2012). Molecular variability in the *Celleporella hyalina* (Bryozoa; Cheilostomata) species complex: Evidence for cryptic speciation from complete mitochondrial genomes. *Molecular Biology Reports*, *39*, 8601–8614. https://doi.org/10.1007/s11033-012-1714-9

Walters, L. J., & Wethey, D. S. (1986). Surface topography influences competitive hierarchies on marine hard substrata: A field experiment. *Biological Bulletin*, *170*(3), 441–449.

Wang, H., Zhang, H., Wong, Y. H., Voolstra, C., Ravasi, T., B Bajic, V., & Qian, P. Y. (2010). Rapid transcriptome and proteome profiling of a non-model marine invertebrate, Bugula neritina. *Proteomics*, *10*(16), 2972–2981.

Wanninger, A., Koop, D., & Degnan, B. M. (2005). Immunocytochemistry and metamorphic fate of the larval nervous system of Triphyllozoon mucronatum (Ectoprocta: Gymnolaemata: Cheilostomata). *Zoomorphology*, *124*(4), 161–170. https://doi.org/10.1007/s00435-005-0004-7

Ward, M. A., & Thorpe, J. P. (1991). Distribution of encrusting bryozoans and other epifauna on the subtidal bivalve *Chlamys opercularis*. *Marine Biology*, *110*, 253–259.

Wasson, B., & De Blauwe, H. (2014). Two new records of cheilostome Bryozoa from British waters. *Marine Biodiversity Records*, *7*.

Waters, A. W. (1879). On the Bryozoa (Polyzoa) of the Bay of Naples. Cheilostomata (continued). *Annals and Magazine of Natural History, Ser. 5*, *3*, 114–126.

Waters, A. W. (1883). On fossil chilostomatous Bryozoa from Muddy Creek, Victoria. *Quarterly Journal of the Geological Society of London*, *39*, 423–443.

Waters, A. W. (1897). Notes on Bryozoa from Rapallo and other Mediterranean localities. - Chiefly Cellulariidae. *Journal of the Linnean Society of London, Zoology*, *26*, 1–23.

Waters, A. W. (1905). Bryozoa from near Cape Horn. *Journal of the Linnean Society (Zoology)*, *29*, 230–251.

Waters, A. W. (1906). Bryozoa from Chatham Island and d'Urville Island, New Zealand, collected by Professor H.Schauinsland. *Annals and Magazine of Natural History*, *(7)17*, 12–23.

Waters, A. W. (1913). The marine fauna of British East Africa and Zanzibar, from collections made by Cyril Crossland, M.A., B.Sc., F.Z.S., In the years 1901-1902. Bryozoa - Cheilostomata. *Proceedings of the Zoological Society of London*, *1913*, 458–537.

Waters, A. W. (1918). Some collections of the littoral marine fauna of the Cape Verde Islands, made by Cyril Crossland, M.A., B.Sc., F.Z.S., In the summer of 1904. - Bryozoa. *Journal of the Linnean Society (Zoology)*, *34*, 1–45.

Waters, A. W. (1925). Some cheilostomatous Bryozoa from Oran (Algiers). *Annals and Magazine of Natural History*, *9*(15), 651–661.

Watts, P. C., & Thorpe, J. P. (2006). Influence of contrasting larval developmental types upon the population-genetic structure of cheilostome bryozoans. *Marine Biology*, *149*,

1093–1101.

Wejnert, K. E., & Smith, A. M. (2008). Within-colony variation in skeletal mineralogy of *Adeonellopsis* sp. (Cheilostomata: Bryozoa) from New Zealand. *New Zealand Journal of Marine and Freshwater Research*, *42*, 389–395.

Wendt, D. E. (1996). Effect of larval swimming duration on success of metamorphosis and size of the ancestrular lophophore in *Bugula neritina* (Bryozoa). *Biological Bulletin*, *191*, 224–233.

Wendt, D. E., & Woollacott, R. M. (1999). Ontogenies of phototactic behavior and metamorphic competence in larvae of three species of *Bugula* (Bryozoa). *Invertebrate Biology*, *118*(1), 75–84. https://doi.org/10.2307/3226915

Wicksten, M.-K. a. (1996). Anthozoans, bryozoans, brachiopods, and tunicates of Rocas Alijos. *Monographiae Biologicae*, *75*, 277–283.

Wieczorek, S. K., & Todd, C. D. (1997). Inhibition and facilitation of bryozoan and ascidian settlement by natural multi-species biofilms: Effects of film age and the roles of active and passive larval attachment. *Marine Biology*, *128*, 463–473.

Winston, J. E. (1984). Shallow-water bryozoans of Carrie Bow Cay, Belize. *American Museum Novitates*, *2799*, 1–38.

Winston, J. E. (1984). Why bryozoans have avicularia: A review of the evidence. *American Museum Novitates*, (2789), 1–26.

Winston, J. E. (2004). Bryozoans from Belize. *Atoll Research Bulletin*, *523*, 1–14.

Winston, J. E. (2009). Stability and change in the Indian River area bryozoan fauna over a twenty-four year period. *Smithsonian Contributions to the Marine Sciences*, *38*, 229–239.

Winston, J. E. (2016). Bryozoa of Floridan Oculina reefs. *Zootaxa*, *4071*(1), 1–81.

Winston, J. E., & Eiseman, N. J. (1980). Bryozoan-algal associations in coastal and continental shelf waters of eastern Florida. *Florida Scientist*, *43*(2), 65–74.

Winston, J. E., & Hakansson, E. (1986). The interstitial bryozoan fauna from Capron Shoal, Florida. *American Museum Novitates*, *2865*, 1–50.

Winston, J. E., & Hayward, P. J. (2011). Bryozoans of the Northeast Coast of the United States: Taxonomic history and summary of a new survey. In P. N. Wyse Jackson & M. E. Spencer Jones (Eds.), *Annals of Bryozoology 3: Aspects of the history of research on bryozoans* (pp. 201–217). Dublin: International Bryozoology Association.

Winston, J. E., & Heimberg, B. F. (1986). Bryozoans from Bali, Lombok, and Komodo. *American Museum Novitates*, *2847*, 1–49.

Winston, J. E., & Maturo, F. J. S. (2009). Bryozoans (Ectoprocta) of the Gulf of Mexico. In D. L. Felder & D. K. Camp (Eds.), *Gulf of Mexico Origin, Waters, and Biota. Volume I, Biodiversity* (pp. 1147–1164). College Station: Texas A&M University Press.

Winston, J. E., & Vieira, L. M. (2013). Systematics of interstitial encrusting bryozoans from southeastern Brazil. *Zootaxa*, *3710*(2), 101–146.

Winston, J. E., Vieira, L. M., & Woollacott, R. M. (2014). Scientific results of the Hassler Expedition. Bryozoa. No. 2. Brazil. *Bulletin of the Museum of Comparative Zoology*, *161*(5), 139–239.

Winston, J. E., & Woollacott, R. M. (2008). Redescription and revision of some red-pigmented *Bugula* species. *Bulletin of the Museum of Comparative Zoology*, *159*(3), 179–212. https://doi.org/10.3099/0027-4100(2008)159%5B179:RAROSR%5D2.0.CO;2

Wong, Y. H., Arellano, S. M., Zhang, H., Ravasi, T., & Qian, P.-Y. (2010). Dependency on de novo protein synthesis and proteomic changes during metamorphosis of the marine bryozoan *Bugula neritina*. *Proteome Science*, *8*(1), 25. https://doi.org/10.1186/1477-5956-8-25

Wood, A. C. L., & Probert, P. K. (2013). Bryozoan-dominated benthos of Otago shelf, New Zealand: Its associated fauna, environmental setting and anthropogenic threats. *Journal of the Royal Society of New Zealand*, *43*(4), 231–249.

Wood, A. C. L., Rowden, A. A., Compton, T. J., Gordon, D. P., & Probert, P. K. (2013). Habitat-forming bryozoans in New Zealand: Their known and predicted distribution in relation to broad-scale environmental variables and fishing effort. *PLoS ONE*, *8*(9), e75160.

Woollacott, R. M., & Zimmer, R. L. (1972a). Fine structure of a potential photoreceptor organ in the larva of *Bugula neritina* (Bryozoa). *Zeitschrift Für Zellforschung Und Mikroskopische Anatomie*, *123*(4), 458–469.

Woollacott, R. M., & Zimmer, R. L. (1972b). Origin and structure of the brood chamber in *Bugula neritina* (Bryozoa). *Marine Biology*, *16*(2), 165–170. https://doi.org/10.1007/BF00347954

Woollacott, R. M., & Zimmer, R. L. (1975). A simplified placenta-like system for the transport of extraembryonic nutrients during embryogenesis of *Bugula neritina* (bryozoa). *Journal of Morphology*, *147*(3), 355–377. Retrieved from http://onlinelibrary.wiley.com/doi/10.1002/jmor.1051470308/abstract

Xing, J., & Qian, P. Y. (1999). Tower cells of the marine bryozoan *Membranipora membranacea*. *Journal of Morphology*, *239*(2), 121–130.

Yang, H. J., Seo, J. E., & Gordon, D. P. (2018a). Sixteen new generic records of Korean Bryozoa from southern coastal waters and Jeju Island, East China Sea: Evidence of tropical affinities. *Zootaxa*, *4422*(4), 493–518. https://doi.org/10.11646/zootaxa.4422.4.3

Yang, H. J., Seo, J. E., Min, B. S., Grischenko, A. V., & Gordon, D. P. (2018b). Cribrilinidae (Bryozoa: Cheilostomata) of Korea. *Zootaxa*, *4377*(2), 216–234. https://doi.org/10.11646/zootaxa.4377.2.4

Yorke, A. F., & Metaxas, A. (2011). Interactions between an invasive and a native bryozoan (*Membranipora membranacea* and *Electra pilosa*) species on kelp and *Fucus* substrates in Nova Scotia, Canada. *Marine Biology*, *158*(10), 2299.

Zabala, M., & Maluquer, P. (1988). Illustrated keys for the classification of Mediterranean Bryozoa. *Treballs Del Museu de Zoologica*, *4*, 1–294.

Zabala i Limousin, M., Maluquer, P., & Harnelin, J. G. (1993). Epibiotic bryozoans on deep-water seleractinian corals from the Catalonia slope (western Mediterranean, Spain, France). *Scientia Marina*, *57*(1), 65–78.

Zabin, C. J., Marraffini, M., Lonhart, S. I., McCann, L., Ceballos, L., King, C., … Ruiz, G. M. (2018). Non-native species colonization of highly diverse, wave swept outer coast habitats in Central California. *Marine Biology*, *165*(2), 31. https://doi.org/10.1007/s00227-018-3284-4

Zabin, C. J., Obernolte, R., Mackie, J. A., Gentry, J., Harris, L., & Geller, J. B. (2010). A non-native bryozoan creates novel substrate on the mudflats in San Francisco Bay. *Marine Ecology Progress Series*, *412*, 129–139.

Zágoršek, K. (1994). Late Eocene (Priabonian) Cheilostomata (Bryozoa) from the Liptov Basin - Western Carpathians (Slovakia). *Neues Jahrbuch Für Geologie Und Paläontologie Monatshefte*, *193*(3), 361–382.

Zágoršek, K., & Vávra, N. (2007). Bryozoan fauna from Steinebrunn (Lower Austria, Badenian) - a revision to establish a basis for comparisons with Moravian faunas. *Scripta Facultatis Scientiarum Naturalium Universitatis Masaryk Brunensis*, *36*, 65–72.

Zenetos, A., Gofas, S., Morri, C., Rosso, A., Violanti, D., García Raso, J. E., … Azzurro, E. (2012). *Alien species in the Mediterranean Sea by 2012. A contribution to the application of European Union's Marine Strategy Framework Directive (MSFD). Part 2. Introduction trends and pathways.*

Zhang, H., Wong, Y. H., Wang, H., Chen, Z., Arellano, S. M., Ravasi, T., & Qian, P. Y. (2011). Quantitative proteomics identify molecular targets that are crucial in larval settlement and metamorphosis of *Bugula neritina*. *Journal of Proteome Research*, *10*(1).

Zimmer, R. L., & Woollacott, R. M. (1989). Larval morphology of the bryozoan *Watersipora arcuata* (Cheilostomata: Ascophora). *Journal of Morphology*, *199*(2), 125–150. Retrieved from http://onlinelibrary.wiley.com/doi/10.1002/jmor.1051990202/abstract

Zongguo, H. (2001). Bryozoa [Ectoprocta]. In *Marine Species and Their Distribution in China's Seas* (pp. 369–383). Malabar: Krieger Publishing Company.
